# Emotions in misinformation studies: distinguishing affective state from emotional response and misinformation recognition from acceptance

**DOI:** 10.1186/s41235-024-00607-0

**Published:** 2024-12-18

**Authors:** Jula Lühring, Apeksha Shetty, Corinna Koschmieder, David Garcia, Annie Waldherr, Hannah Metzler

**Affiliations:** 1 Department of Communication, University of Vienna, Vienna, Austria; 2 Complexity Science Hub, Metternichgasse 8, 1030 Vienna, Austria; 3 Institute of Psychology, University of Graz, Graz, Austria; 4 Center for Research Support, University College for Teacher Education, Graz, Austria; 5 Department of Politics and Public Administration, University of Konstanz, Konstanz, Germany; 6 Institute of Interactive Systems and Data Science, Faculty of Computer Science and Biomedical Engineering, Graz University of Technology, Graz, Austria; 7 Center for Medical Data Science, Medical University of Vienna, Vienna, Austria; 8 Institute for Globally Distributed Open Research and Education, Vienna, Austria

**Keywords:** Misinformation, Emotion, Anger, Discernment, Recognition, Belief, COVID-19

## Abstract

Prior studies indicate that emotions, particularly high-arousal emotions, may elicit rapid intuitive thinking, thereby decreasing the ability to recognize misinformation. Yet, few studies have distinguished prior affective states from emotional reactions to false news, which could influence belief in falsehoods in different ways. Extending a study by Martel et al. (Cognit Res: Principles Implic 5: 1–20, 2020), we conducted a pre-registered online survey experiment in Austria (N = 422), investigating associations of emotions and discernment of false and real news related to COVID-19. We found no associations of prior affective state with discernment, but observed higher anger and less joy in response to false compared to real news. Exploratory analyses, including automated analyses of open-ended text responses, suggested that anger arose for different reasons in different people depending on their prior beliefs. In our educated and left-leaning sample, higher anger was often related to recognizing the misinformation as such, rather than accepting the false claims. We conclude that studies need to distinguish between prior affective state and emotional response to misinformation and consider individuals’ prior beliefs as determinants of emotions.

**Supplementary Information:**

The online version contains supplementary material available at 10.1186/s41235-024-00607-0.

## Introduction

Information processing and beliefs are strongly influenced by emotional and social processes. For instance, we feel threatened when something contradicts beliefs central to our social identity (Robertson et al., 2022; Van Bavel & Pereira, 2018), and can better detect, process, as well as remember emotional information (Mather & Sutherland, 2011). With regard to misinformation specifically, emotions are assumed to foster intuitive thinking, increase reliance on heuristics and familiarity, and distract readers from the quality of information (Ecker et al., 2022). In this way, emotions are thought to reduce deliberation and analytical thinking, and increase belief and sharing of misinformation (Ecker et al., 2022; Martel et al., 2020; Pennycook & Rand, 2021). Indeed, focusing on emotions rather than reason worsens individuals’ discernment between real and false political news items (Martel et al., 2020). Observational analysis of social media data have further found links between the presence of certain emotions in posts and the sharing of rumors (Pröllochs et al., 2021), as well as engagement with both scientific and conspiratorial information (Zollo et al., 2015). Large-scale experimental evidence shows that, in particular, online media articles with negative emotions are shared more often (Robertson et al., 2023). All in all, there seems to be a link between misinformation and emotions, in that such content may be created to maximally evoke emotions, to attract attention, and to trigger belief and sharing of misinformation.

Some findings suggest that a crucial ingredient of emotions in increasing susceptibility to misinformation may be arousal (Berger, 2011; Berger & Milkman, 2013). In particular, high-arousal emotions may boost intuitive thinking and rapid decision-making––leaving people vulnerable to misinformation (Boyer, 2021; Greenstein & Franklin, 2020; Weeks, 2015). According to the theory of core affect, emotions can be characterized along two dimensions: valence, ranging from negative to positive, and arousal, indicating the intensity of the emotion (Russell, 1980). In this study, we analyze valence and arousal as core dimensions of emotions and further focus on two distinct basic emotions due to their relevance in misinformation processing. Anxiety has been linked to increased acceptance of new information (Brader et al., 2008; Weeks, 2015), which could increase susceptibility to misinformation when accurate information is lacking, for example, during a pandemic when scientific studies take longer to be published than false information (Freiling et al., 2021). Anger, on the other hand, is associated with an aversion to new information (MacKuen et al., 2010). Instead, information eliciting anger can increase susceptibility to partisan arguments that confirm a person’s own beliefs (Hasell & Weeks, 2016) and promote the use of heuristics in information processing (Bodenhausen et al., 1994). This may leave individuals susceptible to misinformation based on their existing beliefs (Greenstein & Franklin, 2020; Han et al., 2020). Thus, both anger and anxiety can leave people vulnerable to misinformation in different situations.

Beyond emotion-evoking news or posts, a person’s emotional state also influences how they judge information (Ecker et al., 2022; Schwarz, 2001). Using their general mood as information can influence how people evaluate emotional claims (Kim et al., 2010) or make them vulnerable to persuasion efforts (DeSteno et al., 2004) and misinformation (Greenstein & Franklin, 2020; Weeks, 2015). Martel and colleagues (2020) were the first to show that people reporting general higher emotionality prior to misinformation exposure were more likely to believe false (but not real) news. In the first study of their paper, they observed an association of both negative and positive affective states with a worse ability to discern false from true news. This correlation was significant for all emotion adjectives except those more related to analytic thinking rather than emotionality per se. This correlation was stronger for negative than for positive emotions.

Summarizing the above perspective, emotions overall oppose logical thinking and adversely affect individuals’ judgment (Ecker et al., 2022; Martel et al., 2020), which might in particular apply to negative and high-arousal emotions like anger and fear. However, it is important to note that while this perspective is dominant in misinformation research, there is, so far, only little evidence to support it. Moreover, earlier studies on misinformation have failed to distinguish between a person’s own affective state and emotions elicited by a stimulus, that is, the emotional response to the content. This study therefore distinguishes between a person’s own affective state prior to news exposure, and their response to real and false news stories.

One of our main goals was to test if the association of increased affective state with news discernment, i.e., misinformation acceptance, reported in study 1 by Martel et al. (2020), could be replicated in a different context. Specifically, we tested if the results generalize beyond the American context and to COVID-19-related misinformation. Health misinformation may differ from other (political) misinformation in terms of sentiment and diffusion (Pröllochs et al., 2021) and can be less persistent when not associated with people’s social identity (Vraga et al., 2019; Walter & Murphy, 2018). Nevertheless, due to the political polarization of COVID-19-related health (mis)information and the high emotional involvement with this topic, we expected no substantial differences to the topic of US politics. We therefore expected stronger emotions reported before news exposure to correlate negatively with discernment of false and real news measured in an accuracy rating task (H1). Regarding the valence of emotions, we expected this to hold for both negative (H2a) and positive emotions (H2b), but to be more pronounced for negative emotions (H2c) similar to what Martel and colleagues (2020) find. To help clarify the role of arousal, we measured arousal as a separate dimension of emotion (Russell, 1980). We predicted that arousal would be associated with decreased discernment of news accuracy (H3). Finally, we investigated if specific emotions, like anger (H4) and anxiety (H5) were associated with decreased discernment of real and false news. In addition to the pre-registered hypotheses (https://osf.io/2r6bj), we explored emotional responses to real and false news items, and their relationship with a person's COVID-19 misperceptions.

## Methods

The dataset, code, study materials and pre-registration supporting the conclusions of this article are available in the Open Science Framework repository, https://osf.io/tgzxr/.

### Design

This study was an online survey assessing the relationship between misinformation susceptibility (discernment of news items and agreement with common misperceptions about COVID-19) and emotions. The survey largely followed the design of Martel et al. (2020) but used COVID-19-related news instead of political news. After completing self-reported questionnaires on affective state and agreement ratings for COVID-19 misperceptions, all participants rated the accuracy of real and false COVID-19-related news items (n = 24, within-subject). Additionally, they rated their emotional response to each news item for four basic emotions and expressed their first thoughts in an open-text question.

### Sample

Participants were recruited by university students taking a research seminar, but were not necessarily students themselves. 718 participants started the survey. Only participants who completed the main part of the survey (ending with the news rating items) were included in the analyses. Following the pre-registration, we excluded 248 incomplete responses and 24 underage participants. The reported measures were part of a larger and longer study (see SI for a list of all questionnaires), which took participants a median of 47 min to complete, with 82% needing more than 30 min. To maintain response quality, we removed 24 participants who were too quick (< 20 min). In addition, we checked for repeated response patterns and straightliners, but did not detect any. The final sample contained 422 participants, on average 33.97 years old (*SD* = 15.00); 58.43% identified as female and 40.62% as male. Most participants had completed higher education, with 51.68% having a high school degree and 34.62% having a university degree. Only 4 participants indicated right-wing political orientation. Proportions were: 1.1% right, 8.8% center-right, 21.12% center, 47.59% center-left, and 21.66% left (in total, 36 participants right from center, 259 left from center). See Table S1 for more sample descriptives.

### Procedure

The online survey began with demographic questions, followed by 5 attitude and personality questionnaires (see SI for complete list) only analyzed in student projects. After completing self-reports of affective state during the last few days, participants rated their agreement with 9 statements describing common misperceptions about COVID-19. The news rating task with 12 false and 12 true news items about COVID-19 followed. After each news item, an open-ended question provided the possibility to express any first impulses and thoughts about it. Next, participants rated the accuracy of the news item, the confidence in their judgment, familiarity with the news content, and reported if the item triggered any emotion in them. If this was the case, they rated how much they were experiencing four different basic emotions. After the news rating task, participants completed a few more questions about vaccination status and media use. Participants were debriefed at the end of the survey.

### Measures

#### Independent measures

##### Affective state

Participants reported how they felt in the past few days using the German version of the 20-item Positive and Negative Affect Schedule scale (PANAS; see Breyer & Bluemke, 2016). The PANAS scale does not include all relevant basic emotions, nor enough items that indicate high or low arousal. Therefore, we extended the PANAS scale with 6 additional adjectives (sad, surprised, happy, angry, relaxed, and stressed). Participants rated the intensity with which they experienced each emotion on a 5-point Likert scale ranging from ‘Not at all’ to ‘Extremely’. We analyzed relationships of aggregate positive and negative emotions, arousal as well as individual emotions.

##### Valence

We calculated factor scores via a varimax rotation on a two-factor analysis of all PANAS items, resulting in aggregated scores for positive and negative emotions. For H1, we only examined the original 20 items that were part of the PANAS scale. Specifically, we were looking for overall trends in positive and negative emotions in the PANAS being associated with decreased discernment and accuracy judgments, rather than a specific score. For H2a and H2b, we analyzed the aggregated PANAS scores for all affective state items (original PANAS items + 6 additional adjectives). Whenever we refer to valence in our confirmatory analyses we refer to these extended positive and negative PANAS scores.

##### Arousal

To calculate arousal scores, we took arousal scores for each emotion adjective from the NRC VAD Lexicon (Mohammad, 2018), multiplied them with each participant’s rating for that adjective, and took the mean across these values per participant.

#### Dependent measures

##### COVID-19 misperceptions

We assessed agreement with 9 common misperceptions about COVID-19 (see SI), including vaccine safety and effectiveness (5), nutrition (1), immune response (1), masks (1), and gargle tests (1). An example statement is: “Masks threaten the health of children.” Participants were asked: “On a scale from 0 to 100%, how much do you agree that this statement is true?” After reverse-coding one real statement (all others were false), we calculated an average per participant (*M* = 21.56, *SD* = 19.27, Cronbach’s α = 0.86; see distribution in Fig. S1). The variable was centered around the mean for comparability.

##### News accuracy rating performance

**Fig. 1:**
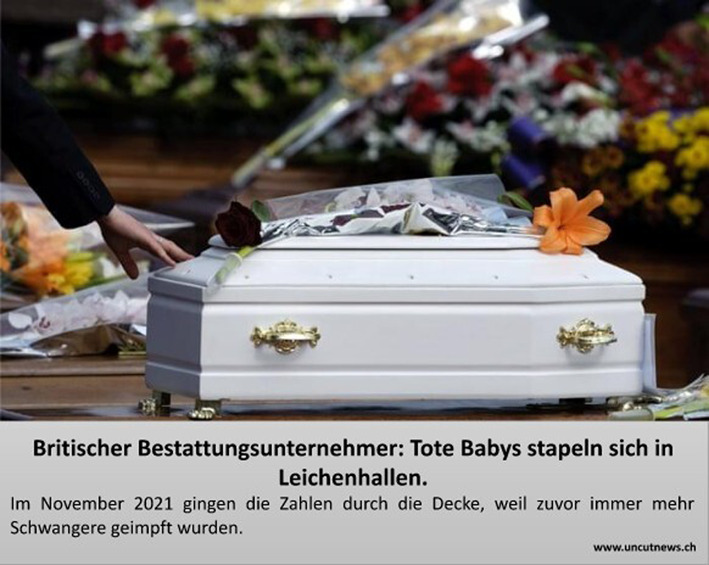
Example news item with headline, teaser, picture and news source

Like in Martel et al. (2020), the 24 news items (12 false, 12 real) resembled a regular social media post with a picture, headline, byline and source (see Fig. 1). False headlines were collected from Austrian and German fact-checking websites about 1–2 months before the survey (www.mimikama.at, https://apa.at/faktencheck/ueberblick/, https://correctiv.org/faktencheck/, https://www.br.de/nachrichten/faktenfuchs-faktencheck), and real news from Austrian mainstream news sites (e.g. DerStandard, Die Presse, Krone). The false items reported on alleged side-effects of preventive healthcare measures and Big Pharma conspiracies. The real items described the benefits and safety of COVID-19 drugs and vaccines, the consequences of vaccine skepticism, updates about new risk factors and virus variants, as well as an initial lack of protective gear. The order of news items was randomized across participants. For each item, participants answered the question: “To the best of your knowledge and belief, how accurate is this news?” on a 0–100% sliding scale (in increments of 10%; initial cursor was set at 50%). We report accuracy rating performance for real (*M* = 67.04, SD = 15.29, Cronbach’s α = 0.80) and false items (*M* = 22.97, SD = 16.24, Cronbach’s α = 0.86), as well as discernment between the two (difference in ratings). The discernment measure was scaled to center around the mean. Distributions of the COVID-19 misperceptions and COVID-19 news discernment are shown in Figure S1.

##### Open-text description of first impulse or thought upon reading a news item

Immediately after each news item, we asked respondents to describe their initial thoughts about it. We introduced this item to capture a raw first mood without focusing on any specific emotions (“What impulse or thought does this news trigger in you?”). In total, there were 5613 textual responses to news items, including 27,468 words (of which 14,224 were in reaction to false and 13,244 to real items). The text was not preprocessed before the analysis with the most recent Linguistic Inquiry and Word Count software (LIWC-2022) using the German dictionary from 2015 (Meier et al., 2019).

##### Emotional responses

After each news item, participants were asked whether they were experiencing any emotion (6-point scale from ‘Not at all’ to ‘Strong’). Participants who reported experiencing an emotion (93.4%) were subsequently asked how much they experienced each of the four basic emotions (anger, anxiety, sadness, joy) or any other emotion. Each emotion was described with 2 adjectives in order to measure each emotion more broadly than with just one specific adjective: upset/angry, frightened/uncertain, sad/concerned, happy/excited. Emotional responses, averaged across news items and participants, were relatively low: *M*_anger_ = 0.93 (SD = 0.94), *M*_anxiety_ = 0.49 (SD = 0.63), *M*_sadness_ = 0.64 (SD = 0.73), *M*_joy_ = 0.28 (SD = 0.47).

### Pilot study

32 students, who also recruited participants later, served as test participants for our study design and items. We examined news rating accuracy for 31 items, agreement with 13 COVID-19-related misperceptions, and five emotional response items. To develop the COVID-19 misperception statements, we followed a strategy similar to Imhoff and Lamberty (2020) and Roozenbeek et al. (2020). In October 2021, we tracked common COVID-related myths debunked on Austrian news websites, such as Profil.at, and students formulated 13 true and false statements based on them. Overall, the study duration was too long. So, we decided to reduce it by removing unclear items based on feedback, and excluding items with a posterior reliability below 0.75, and the following thresholds for discriminatory power: For news items, we set a somewhat lower-than-usual minimal threshold at > 0.25 due to the small sample size, thereby excluding 3 false and 4 real news stimuli. For COVID-19 misperception statements, we removed 4 real items with discriminatory power < 0.30. The number of items for the final data collection was 24 news items and 9 statements with acceptable internal consistencies (> 0.75).

### Statistical analyses

To account for the repeated measurement, and random variance between participants and news items, we used linear mixed-effects models in line with our pre-registration. For confirmatory analyses H1-H5, we predicted accuracy ratings from news type, affective state, and their interaction as fixed effects. As random effects, we used intercepts for participants (1|participant) and news items (1|item ID), and a slope for news type per participant (news type|participant id; see SI for further details). We ran one model for each of the 26 emotion adjectives, and for each aggregate score for positive and negative valence, and arousal. For confirmatory hypotheses H2 to H5, we used Bonferroni-Holm correction for a total of 5 hypotheses (positive, negative, arousal, anger, anxiety) for each dependent variable (news accuracy rating performance, agreement with COVID-19 misperceptions). For this, hypotheses are ordered starting with the smallest p-value, and then compared to the thresholds of α = {0.01, 0.0125, 0.0167, 0.025, 0.05}.

Our exploratory analyses used linear mixed-effects models to examine emotional responses to the news, and their interaction with discernment abilities and commonly held COVID-19 misperceptions. All exploratory models included random intercepts per participant (1|participant). If applicable and models converged, they also included a random intercept per news item (1|item). First, we ran a model *emotional response*~*news type* + *1|participant* + *1|item* for each of the 4 basic emotions to test differences in emotional responses to false and real news. Second, we explored how emotional responses depend on discernment abilities (*emotional response*~accuracy rating*news type + (1*|participants*). Finally, we explored how the emotional response to news differs depending on participants’ agreement with COVID-19 misperceptions (e.g., *anger*~COVID-19 misperceptions * news type + *1|participants*; see SI for details).

### Pre-registration and deviations

The pre-registration is available at https://osf.io/2r6bj. C.K. organized the data collection as part of an undergraduate psychology course in December 2021 at the University of Graz in Austria. She only provided the data to the authors writing the pre-registration after its registration on the OSF on January 20, 2022. The authors received no information about the data prior to the pre-registration.

The pre-registration focused on correlations of affective state prior to news exposure with performance. Beyond correlations with news discernment, H2–H5 were also pre-registered for a second variable, agreement with common COVID-19-related misperceptions (see SI for results). The pre-registration further mentions planned analyses for sadness prior to news exposure, emotional responses to the news items, and previously held misperceptions moderating these reactions. Yet, except for sadness, these analyses were not sufficiently specified (McPhetres, 2020) and are therefore referred to as exploratory.

Regarding deviations, the pre-registration falsely mentions 31 instead of 24 news items and 11 instead of 9 statements about COVID-19 misperceptions (the numbers from the pilot test). We had to omit the planned exploratory analyses on vaccination status and political orientation because there were too few unvaccinated (4.5%) and right-leaning participants (~ 8.5%), and analyses on minority status because a majority of participants (79.6%) considered themselves part of a minority, suggesting that the item was phrased too broadly. Finally, we need to mention that we used the shortest reference time period (‘the last few days’) provided in the default instructions of the German PANAS questionnaire (Breyer & Bluemke, 2016). We only noticed later that Martel et al. (2020) had asked participants about their emotions “at this moment”.

## Results

On average, participants were quite good at accurately judging false (M = 22.97, SD = 16.24) and real news (M = 67.04, SD = 15.29). Their affective state self-reports were neutral, that is, exactly in the middle of the scale (M = 2.49, SD = 0.68 across all emotions). On average, participants do not agree much with common COVID-19 misperceptions (M = 21.56, SD = 19.27 on a scale of 1–100%; see SI for sample distributions of each misperception). Emotional responses to news items were quite low overall (false: M = 0.65, SD = 0.87; real: M = 0.52, SD = 0.60). Tables S2/3 provide descriptive statistics for all main variables.

### Confirmatory analyses: affective state

**Fig. 2:**
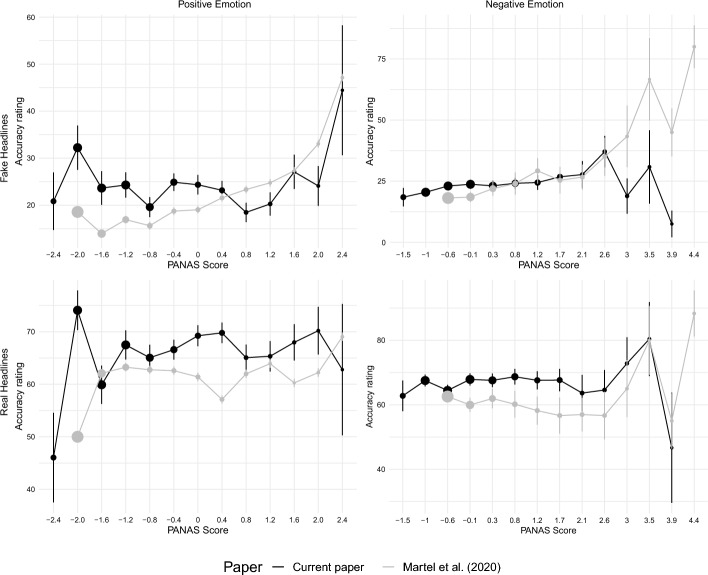
Relationship between news accuracy rating and aggregated positive or negative PANAS scores

In part I, we conceptually replicated Martel et al.’s (2020) analyses on the relationship between affective state and news accuracy ratings (H1). In part 2 (H2-H5), we extended these analyses with additional emotion adjectives, and specifically focused on valence, arousal, anger, and anxiety. Table 1 summarizes these results on affective state, and Table S4 additionally provides 95% CIs, t-values, and standard errors for beta coefficients. Based on Martel et al. (2020)’s results, we predicted increased affective state to correlate with decreased accuracy performance both for false news and discernment (difference false vs. real news). We expected this correlation for aggregate positive and negative scores and most single PANAS adjectives (H1, see https://osf.io/2r6bj). Martel et al. observed 35 significant coefficients in total: Both aggregate scores and 16 out of 20 adjectives significantly and negatively predicted performance for false news and discernment scores, showing that the effect was specific to false news. Overall, our pattern of results did not support this pattern (H1). We only found two significant coefficients in the predicted direction (“jittered”, “afraid”) for false news, and none for discernment. “Inspired” had significant coefficients in the opposite direction. Out of both aggregate PANAS scores, only negative affective state was weakly correlated with false news ratings (b = 1.57, *p* = 0.048), but not with discernment (b = − 0.55, *p* = 0.450). However, this association was not as consistent across adjectives as the correlation between positive or negative affective state and accuracy judgements of false news in Martel et al. (2020) (see Fig. 2). Additionally, when we expanded the aggregate scores with 6 adjectives (H2), this association with negative emotion was no longer significant (b = − 0.55, *p* = 0.488), nor was the one with discernment (b = 1.36, *p* = 0.238). Coefficients for positive emotions with additional adjectives (H2b) were also not significant, nor were those for arousal (H3, false: b = 0.04, *p* = 0.141; real: b = − 0.01, *p* = 0.604; discernment: b = − 0.05, *p* = 0.174).

**Table 1:** Association of affective state ratings with accuracy ratings for each news type (fake, real) and difference in associations between fake and real news (discernment)

	False	Real	Discernment
*Active*	0.37	− 0.50	− 0.87
*Distressed*	1.01	0.17	− 0.84
Interested	− 1.21	0.74	1.95
*Excited*	− 0.85	0.01	− 0.86
*Upset*	0.54	0.81	0.26
*Strong*	− 0.44	0.56	1.00
*Guilty*	0.53	0.79	0.25
*Scared*	1.17	− 0.40	− 1.56
*Hostile*	− 0.78	0.32	1.10
***Inspired***	− 2.41**	1.02	3.43**
*Proud*	0.47	0.07	− 0.40
*Irritable*	1.21	1.38	0.17
*Enthusiastic*	− 0.56	1.20	1.76
*Ashamed*	1.29	0.43	− 0.86
Alert	0.76	− 0.62	− 1.38
*Nervous*	1.59	− 0.25	− 1.84
Determined	− 0.29	0.32	0.62
Attentive	− 0.98	0.64	1.63
*Jittery*	2.12**	0.49	− 1.63
*Afraid/ Anxious*	1.81*	− 0.07	− 1.88
*Positive PANAS*	− 0.68	0.62	1.30
*Negative PANAS*	1.57*	0.70	− 0.87
Sad	0.82	0.30	− 0.52
Surprised	2.23**	0.00	− 2.23
Happy	− 0.81	1.45	2.26
Angry	1.11	0.00	− 1.11
Relaxed	− 0.87	0.77	1.64
Stressed	− 0.30	1.62*	1.92
Positive (extended)	1.52	0.64	− 0.88
Negative (extended)	− 0.55	0.80	1.36

Unstandardized beta coefficients from linear mixed effects models for each emotion: *accuracy* ~ *emotion* + *news type* + *emotion:news type*. False/Real = beta coefficients for the correlation between increased affective state and accuracy ratings for false/real news; Discernment = interaction between emotion and news type

Significance levels: * < .05, ** < .01

**Bold**: Significant discernment effect in this study. Italic: Significant discernment effect in Martel et al. (2020), see Table 1

We had further hypothesized that anger and anxiety decrease accuracy judgments. Yet, coefficients for anger were not significant (H4, false: b = 1.11, *p* = 0.163; real: b = 0.001, *p* = 0.998; discernment: b = − 1.11, *p* = 0.334). In contrast, higher anxiety correlated with rating false news items as more accurate (H5, b = 1.81, *p* = 0.023), but this relationship was not significant after the pre-registered Bonferroni-Holm correction. Anxiety coefficients for real news (b = − 0.07, *p* = 0.925) and discernment (b = − 1.88, *p* = 0.101) were not significant. We repeated all above analyses with COVID-19 misperceptions as a second dependent variable. Again, no consistent correlation pattern emerged between affective state and agreement ratings (see Table S5).

#### Exploratory analyses: responses to false and real news

##### Emotional Responses

**Fig. 3:**
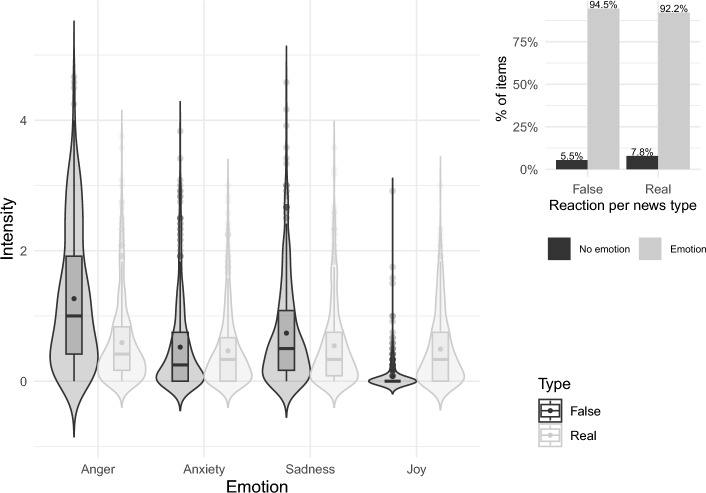
Differences in emotions after fake versus real news exposure. **A** emotion intensity ratings. **B** % of items for which participants indicated feeling an emotion (versus no emotion)

We measured the emotional responses to the news items in addition to the emotional state as a more immediate measure of the emotional involvement. Overall, most participants experienced an emotional reaction to news compared to feeling no emotion (93.4% of participants in total). Compared to feeling no emotion, 94.5% of participants indicated an emotional reaction to false news; similarly, 92.2% participants indicated one to real news (see upper right panel in Fig. 3 for proportions of emotional reactions to fake vs. real news). For all emotional response ratings (upset/angry, frightened/uncertain, sad/concerned, happy/excited), the models converged, but only the model predicting anger from news type showed a significant difference in means, such that anger levels were 0.67 points higher for false than real items (b = 0.44, 95%CI[0.24, 0.64], t = 4.31, *p* < 0.001). On a 6-point scale, this is a substantial difference, and the standardized coefficient also indicates a considerable association. There was a similar but weaker and non-significant pattern for sad/concerned (b = 0.16, [− 0.04, 0.35], t = 1.59, *p* = 0.111) and frightened/uncertain (b = 0.05, [− 0.09, 0.29], t = 0.74, *p* = 0.457), although here, the differences were not significant at the alpha-level of 0.05. Only ratings for joy (happy/excited) were lower for false compared to real items (b = − 0.46, [− 0.75, − 0.18], t = − 3.17, *p* = 0.002).

#### First impulse or thought descriptions

**Fig. 4:**
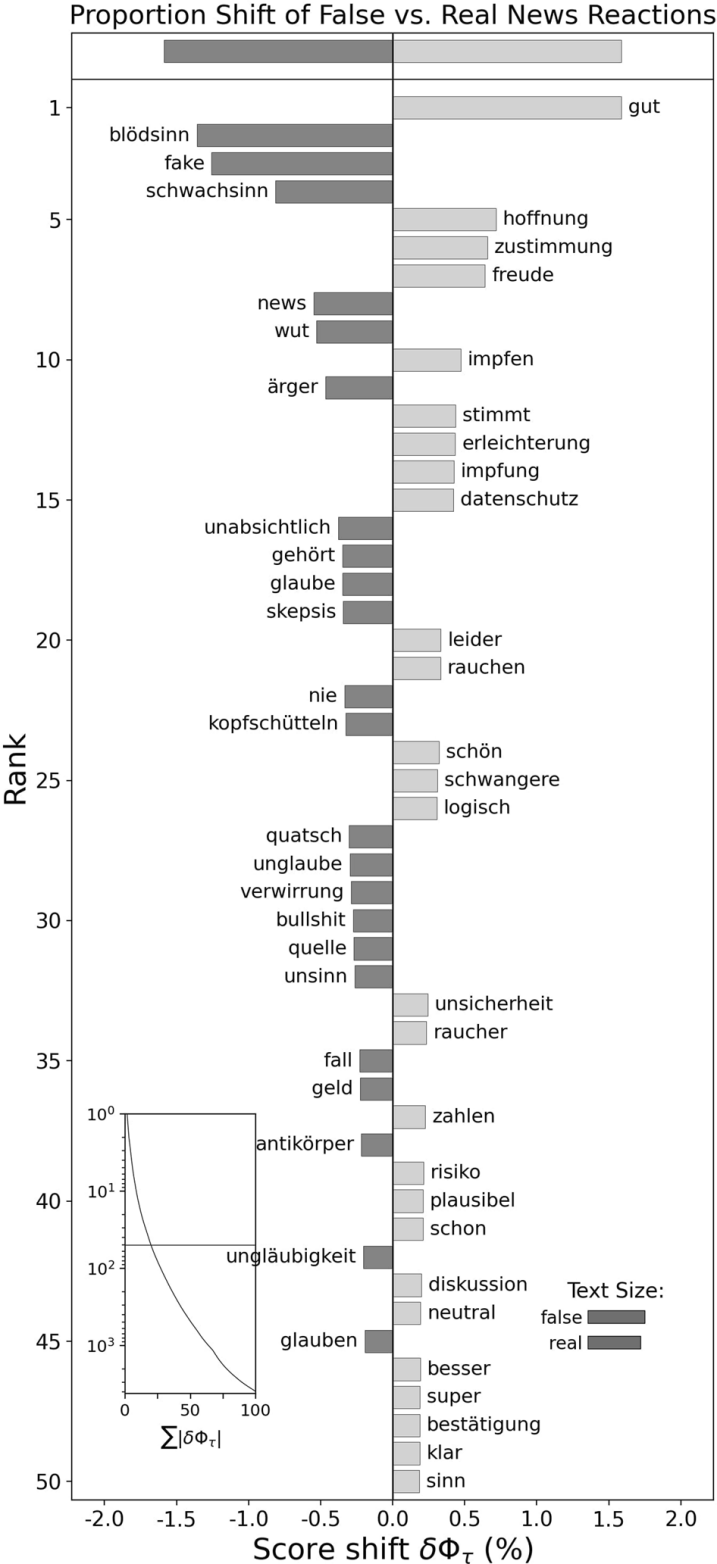
Word shift graph showing differences in text responses describing thoughts and impulses after false compared to real news. *Note* Total *N* = 27,468 in words (5613 texts). The left side (negative score) means words were more frequent after false news (*N* = 14,224), whereas words on the right side were more frequent after real news (*N* = 13,244). Words were translated from German. For original German words, see Figure S4

To get a better understanding of why anger was higher in response to false news while anger was low in the emotional state, we looked at the word frequencies in free-text descriptions. A sentiment analysis with the 2015 German LIWC dictionary showed that out of 27,468 words, 527 words expressed an angry reaction to false news (3.71% of all reactions to false), whereas 141 words indicated an angry reaction to real news (1.06%). Figure 4 further compares the relative frequencies of words in the textual responses to false vs. real news and visualizes the difference in their proportions. Such word shift graphs show which words most strongly contribute to the differences between two texts (Gallagher et al., 2021). Higher ranked words were more frequent after either false or real news, as also indicated by the score shift on the x-axis. Interestingly, the highest ranked words after false news included bullshit (Blödsinn), fake, bollocks/nonsense (Quatsch, Schwachsinn, Unsinn), skepticism (Skepsis), and disbelief (Unglaube), or shaking (my) head (Kopfschütteln), suggesting that many participants recognized the false news and expressed disbelief. Further, some of the highly ranked words expressed anger and irritation (Wut, Ärger, Kopfschütteln). Similar patterns can be observed in Figure S2 (absolute word frequencies) and S2 (word shift graph angry vs. non-angry responses). This suggests that a frequent reason for angry responses to false news was the recognition of such news as false.

#### Emotional response and discernment

**Fig. 5:**
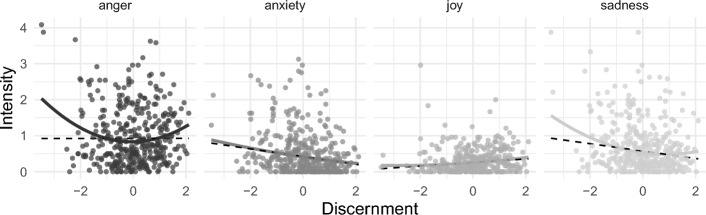
Robust polynomial curves for news discernment and emotional responses. *Note* Black dashed line represents the alternative (robust) linear model

The textual responses to false news indicated that some participants recognized false information and expressed anger related to their disbelief. This could hint at a non-linear relationship between discernment and emotional responses, where stronger anger corresponds to a lower and a higher ability to recognize false headlines. Therefore, we first plotted graphs for the four rated emotions fitting both linear and curvi-linear relationships (see Fig. 5). The scatterplots show a linear positive relationship between anxiety and accuracy ratings for false items (b = 0.21, 95%CI[0.17, 0.24], *p* < 0.001, see Table S6/7 for detailed results), resulting in a negative relationship with discernment (b = − 0.18, [− 0.23, − 0.13], *p* < 0.001). That is, a more anxious response was correlated with lower discernment abilities, i.e., when a false headline made people more anxious, they were more likely to rate it as accurate. Joy also showed a linear and positive relationship, but only for real news (b = 0.24,[0.20, 0.27], *p* < 0.001), and discernment (b = 0.20, [0.15, 0.26] *p* < 0.001; Tables S8, S9). In contrast, anger and sadness plots showed a weak curvi-linear tendency, so we compared mixed-effects models with raw second-order polynomial terms to the linear mixed-effects model for anger (Table S10–S15) and sadness ratings (Tables S16–S21).

We evaluated model fit in two steps: First, we compared polynomial to linear model performance using a chi-square test for nested models, as well as marginal and conditional R^2^s. Second, we assessed the robustness of the curves by excluding potential outliers. For anger, the polynomial model fitted better, χ2(2) = 215.06, *p* < 0.001, and the explained variance increased by 2% (marginal and conditional R2/with and without random effects). This suggests that the polynomial model described the pattern shown in the scatterplot slightly better. In addition, outlier analysis did not reveal any influential cases (Cook’s distance not greater than 0.05; see Table S14 and S15 for models excluding outliers). The first-order (b = − 0.09, [− 0.13, − 0.05], *p* < 0.001) and the non-orthogonalized second-order term (b = 0.29, [0.25, 0.33], *p* < 0.001) indicate a positive (convex) quadratic curve for false items (a = − 0.05, [− 0.11, − 0.02], *p* < 0.001), i.e., both lower and higher levels of discernment were related to higher anger, while moderate discernment abilities co-occured with lower anger scores. In other words, people who were especially good or bad at recognizing false news were more angry after exposure to the false news. For sadness, the polynomial model also seemed to describe the data slightly better, χ2(2) = 24.78, *p* < 0.001, but the fixed effects for false items were very small, explaining less than 1% of variance more. Therefore, the simpler linear model seemed to fit better (for false items: b = 0.11, 95%CI[0.08, 0.15], *p* < 0.001; for real items: b = 0.08,[0.04, 0.11], *p* < 0.001; see Tables S16 and S18).

#### Emotional responses and agreement with COVID-19 misperceptions

**Fig. 6:**
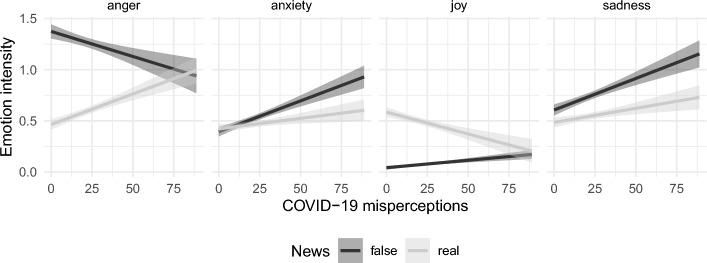
Emotional reaction intensity per news type in relation to COVID-19 misperceptions

Lastly, to understand if agreement with COVID-19 misperceptions similarly explains the angry responses to false news items, we ran a linear mixed-effects model on each of the four emotional responses. Full results are reported in Table S22 and visualized in Fig. 6. When exposed to real news items, participants with who agreed more with COVID-19 misperceptions reported more anger (b = 0.12, 95% CI [0.04, 0.19], *p* < 0.001) and less joy (b = − 0.08, [− 0.12, − 0.05], *p* < 0.001) than participants who agreed less with these misperceptions. Anxiety and sadness responses to real news were not significantly correlated with COVID-19 misperceptions (anxiety: b = 0.04, [− 0.02, 0.10], *p* = 0.190; sadness: b = 0.05, [− 0.01, 0.12], *p* = 0.124). In contrast, participants who agreed more with these misperceptions also reported higher anxiety (b = 0.12, [0.06, 0.17], *p* < 0.001) and sadness (b = 0.12, [0.05, 0.19], *p* < 0.001) after false news, as well as slightly lower angry responses (b = − 0.09, [− 0.17, − 0.02], *p* = 0.020). In brief, higher agreement with COVID-19 misperceptions contributed to more angry and less happy responses to real news, and more anxious and sad responses to false news.

## Discussion

To better understand the role of emotions in misinformation processing, we conducted an online survey in Austria in December 2021. Specifically, we assessed the negative correlation between prior affective state and discernment between false and real news items about COVID-19. Our study conceptually followed a correlational study from Martel et al. (2020), which found that stronger emotions before misinformation exposure coincide with a lower performance in discerning false from real news. We extended this study in multiple ways: First, we distinguished prior affective state from direct emotional responses to news items; second, we zoomed in on arousal and valence in addition to specific emotions like anger and anxiety. Third, we used COVID-19-related misinformation instead of political misinformation; finally, we gave participants the opportunity to describe their first thoughts about news items.

In contrast to Martel et al.’s (2020) study 1, we could not confirm a correlation of affective state overall (H1), or positive (H2a) and negative (H2b) affective state separately, with misinformation discernment. Concretely, people were neither worse at discerning real from false news when they reported stronger emotions prior to exposure, nor did they agree more with common misperceptions about COVID-19 vaccines and other preventive behaviors. We pre-registered these hypotheses based on prior literature, which suggested that emotions worsen misinformation discernment, with evidence seeming most robust for high-arousal emotions (H3), including particularly anger (H4) and anxiety (H5). Yet, our analyses did not consistently support any of these pre-registered hypotheses about the prior affective state. Methodological differences between the two studies might partially explain this and we discuss their implications below. More importantly, however, these null-findings confirmed initial reservations we held when pre-registering our hypotheses based on Martel et al.’s results: We found it more plausible that the immediate emotions elicited by the news (rather than a person’s potentially unrelated general affective state) would be linked to their accuracy judgments. We therefore discuss reasons for this together with our exploratory findings in the next paragraph.

Our planned exploratory analyses on participants’ emotional response to false and real news items showed that participants reacted with higher anger for false and higher joy ratings for real news items. First, a simple explanation for higher joy responses after real news in our study is that half of these items reported on the safety and benefits of vaccines. In our sample, where only 4.5% of all participants said they did not intend to get vaccinated, these news items likely elicited positive emotions. Second, regarding angry reactions to false news, participants’ first thought descriptions were insightful: Compared to real news, they more often contained words expressing anger and words like *bullshit*, *nonsense*, *rubbish, fake, skepticism,* or *disbelief.* These words reveal that most participants were not angry because they believed the false news stories but because they recognized them as false. In line with this, we observed a non-linear relationship between angry responses and discernment: both people with high and low discernment abilities reported higher anger than people with average discernment. This indicates that anger arose for different reasons across participants with different existing beliefs: Some may have shown anger *because* of the misinformation, but most seem to have expressed their anger *about* the misinformation. Anger did not make people more susceptible to misinformation per se; rather, it seemed to depend on their interpretation, and thus, the congruence with their existing views. Further exploratory analyses confirmed that participants with stronger misperceptions about COVID-19 were more angry and less happy after real news and less angry about false news. These participants also reported feeling more anxiety and sadness after reading false news, which makes sense if they believed their content. That anger after false news in our study seems to mainly indicate recognition, and not acceptance, is consistent with a recent study in which participants with higher discernment abilities reported anger more often, but only for politically discordant headlines (Bago et al., 2022). Together with this, our results suggest that factors like (political) beliefs and worldviews may be crucial for understanding the association between emotion and perceived news accuracy.

Overall, our results are not consistent with the idea that emotions simply enhance susceptibility to misinformation. Instead, most people who experienced anger after false news did not accept the news items’ false claims, but were skeptical of them. On the contrary, people who accepted false news got angry after real news, and tended to have stronger misperceptions. This pattern of results is consistent with the concept of “open vigilance” (Mercier, 2021; Sperber et al., 2010). Vigilance, here, suggests that people are skeptical by default as an evolutionary defense mechanism against bad-faith actors. If emotions always increased susceptibility to information, bad-faith actors would consistently use emotions to deceive and exploit individuals. This is not evolutionarily adaptive. Instead, people only *reduce* their vigilance and become *open* to accept new information if it passes several trustworthiness checks (Mercier, 2021; Sperber et al., 2010). For instance, individuals check if new information fits with their existing knowledge, comes from a trusted source, or includes good arguments (Mercier, 2017, 2021). Therefore, when conceptualizing emotions as communicative signals within such a framework (Dezecache & Mercier, 2022), we need to consider the adaptive function of emotions in a given situation to understand their role, and potential effects on news judgments.

Our results suggest that emotions can have beneficial effects on misinformation discernment in some cases, rather than making people more gullible in general (Holland et al., 2012; Kaplan et al., 2016; Van Damme & Smets, 2014; Yeo & McKasy, 2021). In some cases, emotions may reinforce existing misperceptions, and increase acceptance of inaccurate information that is congruent with our beliefs (Bago et al., 2020; Knobloch-Westerwick et al., 2020; Wischnewski & Krämer, 2021). Yet, when misinformation contradicts existing beliefs, an angry reaction may also help us recognize implausible claims. So to understand this differential function of emotion in news discernment, we need to distinguish between emotions already present in the person from those that the stimulus elicits (Van Damme & Smets, 2014), and consider the reason for the emotion, which often relates to existing beliefs (Ecker et al., 2022). The source of an emotion can hint at potential reasons. In our case, observing an association of news discernment with emotional responses, but not affective state, suggests that the content of news stimuli (together with existing beliefs) determined the meaning of the emotion.

Beyond these theoretical arguments, methodological differences of our study with Martel et al.’s (2020) study 1 might partially explain null-results for affective state (H1-H5). First, we asked participants to rate their emotional experience over the past few days, whereas Martel et al. (2020) asked about emotions at the moment. We only noticed this difference after data collection. Nevertheless, we observed significant variance in emotions, and consider it likely that participants relied in part on their current affective state when trying to recall their emotions during the last days (Robinson & Clore, 2002; Van Boven et al., 2009). If one assumes that asking about a slightly longer time period explains our null-result for affective state, this would still support the argument that the more temporally proximate an emotion is to misinformation exposure, the more relevant it is. In other words, immediate emotional responses are more relevant than current affective state, which is more relevant than the affective state during the last days. Second, asking participants to rate their emotions after each news item could potentially have weakened the association of affective state and news discernment. Both because it increased temporal distance between the affective state self-reports and later news items, and because labeling an emotion can reduce the intensity of emotional experiences.

Third, results may differ due to our non-representative sample, which was recruited by psychology students (but not composed of students alone), and therefore, was more left-leaning and highly educated. The meaning of an emotional response to news differs across individuals depending on their beliefs. In 2021, people in Austria on the political far-right were generally opposed to COVID-19 measures and protective behaviors, and thus, potentially more likely to fall for COVID-19 misinformation. While we didn’t have many participants from the far-right, we still observed considerable variance in news discernment, existing misperceptions, and affective state in our left-leaning sample. Hence, we should have been able to observe a correlation between discernment and emotions. Finally, factors like the country and topic difference do not seem like plausible explanations to us. COVID-19 news was as politically polarized in Austria as political news is in the US. Further, given that health is personally relevant to everyone, it is implausible to hypothesize a negative relationship between emotion and misinformation discernment only for political but not COVID-19-related news.

Taken together, we find it unlikely that the methodological differences between our and Martel et al.’s exploratory study 1 (2020) can fully account for the current null-result on affective state and misinformation discernment (H1–H5). Instead, we argue that the evidence from this pre-registered study should be weighted more strongly than the evidence from the earlier exploratory study. At the very least, the current study suggests that emotions do not generally hinder misinformation discernment but that people’s beliefs, the emotion in the content, and the timing of an emotion are crucial factors to consider. In addition to the aforementioned limitations, it is crucial to emphasize that our study does not allow causal conclusions regarding the effects of emotions on misinformation processing. It only allows causal attribution of emotional responses to news items. Regarding more general limitations, the low levels of emotions and high discernment performances in online survey studies like ours (Bago et al., 2022; Martel et al., 2020) point to the compromised ecological validity of study designs based on forced exposure to news items. In addition, the order of the items after news exposure, which involved rating accuracy before emotional responses, might have dampened emotional responses. We still observed sufficient variance and significant results, and a correspondence with open-ended descriptions of initial thoughts and impulses upon reading the news.

Our results highlight that future misinformation studies on emotions should consider measuring immediate emotional responses to news exposure rather than the general affective state, and assessing the reason for experiencing an emotion, especially the existing beliefs. Future studies could further investigate if our findings relying on a measure of news accuracy generalize to other ways of assessing the impact of misinformation (Pennycook & Rand, 2021).

## Conclusion

Increased emotions are not generally associated with increased acceptance of misinformation. Rather, this relationship depends on the function of a specific emotion for a particular person in a particular situation. Our study highlights two factors that can determine the function of emotion with regard to misinformation: the source of the emotion (the general affective state of the person at the moment vs. the emotional response to the information) and the congruence of the person’s general or issue-specific beliefs with the stimuli, which crucially determines the interpretation of the information. To capture such complex relationships, the measurement of emotions is crucial: While the affective state of a person may influence their motivation to seek out new information and their general openness to select relevant information, the immediate emotional reaction to misinformation is more indicative of the emotional processes that influence how participants respond to it. Understanding the emotional processes around belief and sharing of misinformation requires taking into account their function for an individual in a particular context—sometimes, veracity is central to reaching a certain goal, but at other times, it may simply come secondary.

## Supplementary Information

Additional file1 (DOCX 1213 KB)

## Data Availability

The dataset, code, study materials and pre-registration supporting the conclusions of this article are available in the Open Science Framework repository, https://osf.io/tgzxr/
