# Supplementary material for: Emotions in misinformation studies: distinguishing affective state from emotional response and misinformation recognition from acceptance
Source: Cogn Res Princ Implic. 2024 Dec 18;9:82. doi: 10.1186/s41235-024-00607-0 (PMC11656008; doi:10.1186/s41235-024-00607-0)
Supplement: Supplementary file 1 — Additional file1 (DOCX 1213 KB) [file 41235_2024_607_MOESM1_ESM.docx]

Supplementary Information

Emotions in misinformation studies: Distinguishing affective state from emotional response and misinformation recognition from acceptance

Table of contents

[Sample Descriptives 2](#_heading=h.1fob9te)

[Descriptives for Main Variables 2](#_heading=h.r22am9aq2k88)

[Affective State 4](#_heading=h.9y9nzmqe6yb7)

[Statistical analysis: Random effects structure 4](#_heading=h.4xlffn7xs0pa)

[Results 5](#_heading=h.8adf0y2d8v5j)

[Results: Text Responses to News Items 10](#_heading=h.3dy6vkm)

[Results: Emotional Responses and Rating Task Performance: Linear and Curvi-linear (Polynomial) Mixed Effect Models 14](#_heading=h.1t3h5sf)

[Anxiety 14](#_heading=h.4d34og8)

[Joy 15](#_heading=h.17dp8vu)

[Anger 16](#_heading=h.3rdcrjn)

[Anger with excluded outliers 17](#_heading=h.26in1rg)

[Sadness 18](#_heading=h.lnxbz9)

[Emotional Responses and Agreement with False Beliefs about COVID-19 21](#_heading=h.35nkun2)

[Statistical Analysis 21](#_heading=h.jxlfb1fzl09u)

[Questionnaires, Tasks, and Text of Items 21](#_heading=h.1ksv4uv)

[Order of questionnaires and tasks 21](#_heading=h.44sinio)

[Socio-demographics 22](#_heading=h.2jxsxqh)

[Agreement with false COVID-19 Beliefs 23](#_heading=h.z337ya)

[COVID-19 news rating task and emotional response 23](#_heading=h.3j2qqm3)

[Vaccination status 24](#_heading=h.1y810tw)

[References 25](#_heading=h.4i7ojhp)

## Sample Descriptives

**Table S1**

*Distribution of additional demographic variables.*

|  | Frequencies | Proportions |
| --- | --- | --- |
| Minority status |  |  |
| yes | 336 | 79.62% |
| no | 86 | 20.38% |
| N/A | 0 | 0% |
| Vaccination status |  |  |
| yes | 323 | 76.54% |
| no | 19 | 4.50% |
| N/A | 80 | 18.96% |
| Political orientation |  |  |
| left | 81 | 19.19% |
| left-leaning | 178 | 42.18% |
| center | 79 | 18.72% |
| right-leaning | 32 | 7.58% |
| right | 4 | 0.95% |
| N/A | 48 | 11.37% |
| Total *N* | 422 | 100% |

**Figure S1**

*Panel A shows the mean-centered distribution of COVID-19 discernment (where a negative score means low discernment skills). Panel B shows the distribution of prior COVID-19 misperceptions (where a negative score means low misperceptions).*

## *
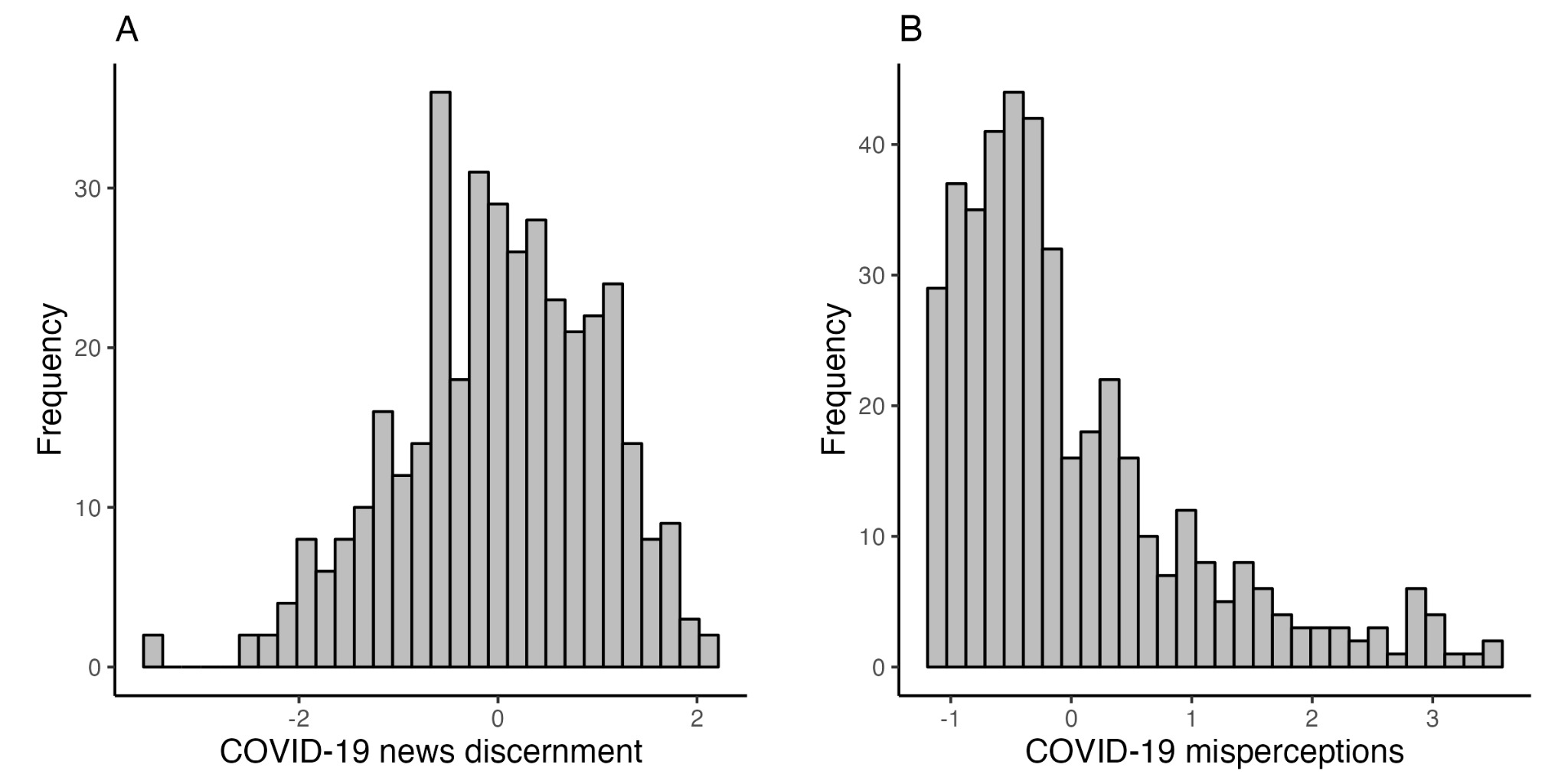
*

## Descriptives for Main Variables

**Table S2**

*Descriptives of main variables*

|  | Mean | SD |
| --- | --- | --- |
| Affective state |  |  |
| Positive | 3.1 | 0.7 |
| Negative | 1.89 | 0.65 |
| Positive (extended) | 3.03 | 0.65 |
| Negative (extended) | 1.95 | 0.67 |
| Anxiety | 1.74 | 0.96 |
| Anger | 1.80 | 1.01 |
| All Emotions (PANAS) | 2.5 | 0.7 |
| All Emotions (Extended) | 2.49 | 0.68 |
| Arousal | 49.91 | 27.69 |
| Accuracy ratings (0-100) |  |  |
| Real News | 67.04 | 15.29 |
| False News | 22.97 | 16.24 |
| False COVID-19 beliefs (0-100) | 21.56 | 19.27 |
| Emotional responses (0-5) |  |  |
| Upset/Angry (false) | 1.27 | 1.06 |
| Upset/Angry (real) | 0.59 | 0.65 |
| Frightened/Uncertain (false) | 0.52 | 0.69 |
| Frightened/Uncertain (real) | 0.47 | 0.56 |
| Sad/Concerned (false) | 0.74 | 0.80 |
| Sad/Concerned (real) | 0.54 | 0.63 |
| Happy/Excited (false) | 0.07 | 0.24 |
| Happy/Excited (real) | 0.49 | 0.54 |
| *N* = 422 |  |  |

**Table S3**

*Descriptives for all PANAS (extended) items*

| Affective State (1-5) | Mean | SD |
| --- | --- | --- |
| Active | 3.26 | 1.02 |
| Distressed | 2.28 | 1.04 |
| Interested | 3.57 | 0.95 |
| Excited | 3.06 | 1.11 |
| Upset | 2.25 | 1.07 |
| Strong | 2.94 | 1.07 |
| Guilty | 1.56 | 0.92 |
| Scared | 1.51 | 0.87 |
| Hostile | 1.44 | 0.79 |
| Inspired | 2.72 | 1.04 |
| Proud | 2.77 | 1.16 |
| Irritable | 2.26 | 1.1 |
| Enthusiastic | 3.06 | 1.16 |
| Ashamed | 1.44 | 0.8 |
| Alert | 3.09 | 0.95 |
| Nervous | 2.14 | 1.14 |
| Determined | 3.15 | 1.03 |
| Attentive | 3.42 | 0.91 |
| Jittery | 2.27 | 1.23 |
| Afraid/Anxious | 1.74 | 0.96 |
| Sad | 2.01 | 1.15 |
| Surprised | 1.88 | 0.99 |
| Happy | 3.45 | 1 |
| Angry | 1.8 | 1.01 |
| Relaxed | 2.98 | 1.08 |
| Stressed | 2.68 | 1.25 |
| All Emotions | 2.49 | 0.68 |

## Affective State

### Statistical analysis: Random effects structure

We used a mixed-effects model to investigate the relationship between accuracy ratings of the news items (dependent variable), affective state, and news type (independent variables). This type of model allows to account for the interdependency between multiple observations from the same participant, as well as for participant- and item-specific variations.

Our initial model was based on the work by Martel and colleagues (2020). As predictors, we entered the affective state rating, the type of news headline (false vs real), and the interaction between the two as fixed effects into the model. Two random intercepts were incorporated to account for variation between news items and participants, we included random intercepts for each predictor. To allow for variation in relationships with performance, we further included random slopes for affective state per news item, and for news type per participant. However, this model with the maximal random effects structure did not converge due to minimal between-item variance for the effect of emotion. We therefore simplified it by removing the by-item random slope for the effect of emotion. Note that the models from Martel and colleagues' paper (2020) also show issues with non-convergence, though they do not correct for it. The formula for the final model structure, including both random intercepts and one random slope for news type, was, therefore: accuracy rating performance ~ affective state * news type + (1 | item ID) + (1| participant) + (news type | participant id).

The baseline for news type was set to “real”, that is, real was coded as 0 and false as 1. Thus, beta coefficients indicate the correlation for false news. To calculate the beta coefficient for real news, we re-leveled the news type variable to make ‘false’ the baseline level, and re-ran each model.

### Results

**Table S4**

*Association of affective state ratings with accuracy ratings for each news type (fake, real), and difference in associations between fake and real news (discernment)*

| **Emotion** | **Association with emotion** | **Coefficient** | **SE** | **95% CI** | **t** | ***p*** |
| --- | --- | --- | --- | --- | --- | --- |
| Active | Fake | 0.37 | 0.80 | [-1.19, 1.94] | 0.47 | 0.641 |
|  | Real | -0.50 | 0.75 | [-1.97, 0.97] | -0.67 | 0.505 |
|  | Discernment | -0.87 | 1.15 | [-3.13, 1.38] | -0.76 | 0.448 |
|  |  |  |  |  |  |  |
| Distressed | Fake | 1.01 | 0.80 | [-0.55, 2.58] | 1.27 | 0.206 |
|  | Real | 0.17 | 0.75 | [-1.3, 1.65] | 0.23 | 0.818 |
|  | Discernment | -0.84 | 1.15 | [-3.1, 1.42] | -0.73 | 0.467 |
|  |  |  |  |  |  |  |
| Interested | Fake | -1.21 | 0.80 | [-2.77, 0.36] | -1.51 | 0.130 |
|  | Real | 0.74 | 0.75 | [-0.73, 2.21] | 0.99 | 0.324 |
|  | Discernment | 1.95 | 1.15 | [-0.3, 4.2] | 1.70 | 0.090 |
|  |  |  |  |  |  |  |
| Excited | Fake | -0.85 | 0.80 | [-2.41, 0.72] | -1.06 | 0.288 |
|  | Real | 0.01 | 0.75 | [-1.46, 1.48] | 0.01 | 0.990 |
|  | Discernment | 0.86 | 1.15 | [-1.4, 3.11] | 0.74 | 0.457 |
|  |  |  |  |  |  |  |
| Upset | Fake | 0.54 | 0.80 | [-1.01, 2.1] | 0.69 | 0.493 |
|  | Real | 0.81 | 0.75 | [-0.65, 2.27] | 1.08 | 0.279 |
|  | Discernment | 0.26 | 1.15 | [-1.98, 2.51] | 0.23 | 0.819 |
|  |  |  |  |  |  |  |
| Strong | Fake | -0.44 | 0.80 | [-2.02, 1.13] | -0.55 | 0.581 |
|  | Real | 0.56 | 0.75 | [-0.91, 2.03] | 0.75 | 0.456 |
|  | Discernment | 1.00 | 1.15 | [-1.26, 3.27] | 0.87 | 0.384 |
|  |  |  |  |  |  |  |
| Guilty | Fake | 0.53 | 0.80 | [-1.03, 2.1] | 0.67 | 0.504 |
|  | Real | 0.79 | 0.75 | [-0.68, 2.26] | 1.05 | 0.293 |
|  | Discernment | 0.25 | 1.15 | [-2, 2.51] | 0.22 | 0.825 |
|  |  |  |  |  |  |  |
| Scared | Fake | 1.17 | 0.79 | [-0.39, 2.72] | 1.47 | 0.142 |
|  | Real | -0.40 | 0.75 | [-1.86, 1.07] | -0.53 | 0.594 |
|  | Discernment | -1.56 | 1.14 | [-3.81, 0.68] | -1.37 | 0.172 |
|  |  |  |  |  |  |  |
| Hostile | Fake | -0.78 | 0.80 | [-2.34, 0.78] | -0.98 | 0.328 |
|  | Real | 0.32 | 0.75 | [-1.14, 1.79] | 0.43 | 0.665 |
|  | Discernment | 1.10 | 1.15 | [-1.15, 3.35] | 0.96 | 0.337 |
|  |  |  |  |  |  |  |
| Inspired | Fake | -2.41 | 0.79 | [-3.96, -0.85] | -3.04 | 0.002 |
|  | Real | 1.02 | 0.75 | [-0.45, 2.49] | 1.36 | 0.175 |
|  | Discernment | 3.43 | 1.14 | [1.19, 5.67] | 3.00 | 0.003 |
|  |  |  |  |  |  |  |
| Proud | Fake | 0.47 | 0.80 | [-1.1, 2.03] | 0.59 | 0.557 |
|  | Real | 0.07 | 0.75 | [-1.4, 1.54] | 0.09 | 0.924 |
|  | Discernment | -0.40 | 1.15 | [-2.65, 1.86] | -0.35 | 0.730 |
|  |  |  |  |  |  |  |
| Irritable | Fake | 1.21 | 0.79 | [-0.34, 2.77] | 1.53 | 0.127 |
|  | Real | 1.38 | 0.75 | [-0.08, 2.85] | 1.86 | 0.063 |
|  | Discernment | 0.17 | 1.15 | [-2.08, 2.42] | 0.15 | 0.881 |
|  |  |  |  |  |  |  |
| Enthusiastic | Fake | -0.56 | 0.80 | [-2.13, 1.02] | -0.69 | 0.489 |
|  | Real | 1.20 | 0.75 | [-0.27, 2.68] | 1.60 | 0.111 |
|  | Discernment | 1.76 | 1.15 | [-0.51, 4.02] | 1.52 | 0.128 |
|  |  |  |  |  |  |  |
| Ashamed | Fake | 1.29 | 0.79 | [-0.26, 2.85] | 1.64 | 0.102 |
|  | Real | 0.43 | 0.75 | [-1.03, 1.89] | 0.58 | 0.562 |
|  | Discernment | -0.86 | 1.14 | [-3.1, 1.38] | -0.75 | 0.451 |
|  |  |  |  |  |  |  |
| Alert | Fake | 0.76 | 0.80 | [-0.81, 2.33] | 0.95 | 0.340 |
|  | Real | -0.62 | 0.75 | [-2.09, 0.85] | -0.83 | 0.407 |
|  | Discernment | -1.38 | 1.15 | [-3.64, 0.87] | -1.20 | 0.229 |
|  |  |  |  |  |  |  |
| Nervous | Fake | 1.59 | 0.80 | [0.03, 3.15] | 1.99 | 0.046 |
|  | Real | -0.25 | 0.75 | [-1.73, 1.22] | -0.34 | 0.734 |
|  | Discernment | -1.84 | 1.15 | [-4.1, 0.41] | -1.60 | 0.109 |
|  |  |  |  |  |  |  |
| Determined | Fake | -0.29 | 0.79 | [-1.85, 1.26] | -0.37 | 0.711 |
|  | Real | 0.32 | 0.75 | [-1.14, 1.79] | 0.43 | 0.664 |
|  | Discernment | 0.62 | 1.14 | [-1.62, 2.86] | 0.54 | 0.589 |
|  |  |  |  |  |  |  |
| Attentive | Fake | -0.98 | 0.80 | [-2.55, 0.59] | -1.23 | 0.219 |
|  | Real | 0.64 | 0.75 | [-0.84, 2.12] | 0.85 | 0.394 |
|  | Discernment | 1.63 | 1.15 | [-0.64, 3.89] | 1.41 | 0.159 |
|  |  |  |  |  |  |  |
| Jittery | Fake | 2.12 | 0.79 | [0.56, 3.67] | 2.67 | 0.008 |
|  | Real | 0.49 | 0.75 | [-0.98, 1.96] | 0.66 | 0.512 |
|  | Discernment | -1.63 | 1.15 | [-3.88, 0.63] | -1.42 | 0.157 |
|  |  |  |  |  |  |  |
| Afraid/ Anxious | Fake | 1.81 | 0.79 | [0.25, 3.36] | 2.28 | 0.023 |
|  | Real | -0.07 | 0.75 | [-1.54, 1.4] | -0.09 | 0.925 |
|  | Discernment | -1.88 | 1.15 | [-4.12, 0.37] | -1.64 | 0.101 |
|  |  |  |  |  |  |  |
| Sad | Fake | 0.82 | 0.80 | [-0.74, 2.38] | 1.03 | 0.302 |
|  | Real | 0.30 | 0.75 | [-1.16, 1.76] | 0.40 | 0.687 |
|  | Discernment | -0.52 | 1.15 | [-2.77, 1.73] | -0.45 | 0.650 |
|  |  |  |  |  |  |  |
| Surprised | Fake | 2.23 | 0.80 | [0.66, 3.79] | 2.79 | 0.005 |
|  | Real | 0.00 | 0.76 | [-1.48, 1.48] | 0.00 | 0.999 |
|  | Discernment | -2.23 | 1.15 | [-4.49, 0.04] | -1.93 | 0.054 |
|  |  |  |  |  |  |  |
| Happy | Fake | -0.81 | 0.80 | [-2.38, 0.76] | -1.01 | 0.313 |
|  | Real | 1.45 | 0.75 | [-0.02, 2.93] | 1.94 | 0.053 |
|  | Discernment | 2.26 | 1.15 | [0.01, 4.52] | 1.97 | 0.049 |
|  |  |  |  |  |  |  |
| Angry | Fake | 1.11 | 0.80 | [-0.45, 2.68] | 1.39 | 0.163 |
|  | Real | 0.00 | 0.75 | [-1.47, 1.47] | 0.00 | 0.998 |
|  | Discernment | -1.11 | 1.15 | [-3.37, 1.14] | -0.97 | 0.334 |
|  |  |  |  |  |  |  |
| Relaxed | Fake | -0.87 | 0.81 | [-2.45, 0.72] | -1.08 | 0.282 |
|  | Real | 0.77 | 0.76 | [-0.71, 2.26] | 1.02 | 0.308 |
|  | Discernment | 1.64 | 1.16 | [-0.64, 3.92] | 1.41 | 0.158 |
|  |  |  |  |  |  |  |
| Stressed | Fake | -0.30 | 0.80 | [-1.87, 1.27] | -0.38 | 0.704 |
|  | Real | 1.62 | 0.75 | [0.15, 3.08] | 2.16 | 0.031 |
|  | Discernment | 1.92 | 1.15 | [-0.33, 4.18] | 1.67 | 0.095 |
|  |  |  |  |  |  |  |
| Positive | Fake | -0.68 | 0.80 | [-2.25, 0.88] | -0.85 | 0.393 |
|  | Real | 0.62 | 0.75 | [-0.85, 2.09] | 0.82 | 0.411 |
|  | Discernment | 1.30 | 1.15 | [-0.96, 3.56] | 1.13 | 0.259 |
|  |  |  |  |  |  |  |
| Negative | Fake | 1.57 | 0.79 | [0.01, 3.12] | 1.98 | 0.048 |
|  | Real | 0.70 | 0.75 | [-0.76, 2.17] | 0.94 | 0.349 |
|  | Discernment | -0.87 | 1.15 | [-3.11, 1.38] | -0.76 | 0.450 |
|  |  |  |  |  |  |  |
| Positive (Extended) | Fake | 1.52 | 0.79 | [-0.04, 3.08] | 1.91 | 0.056 |
|  | Real | 0.64 | 0.75 | [-0.83, 2.11] | 0.86 | 0.391 |
|  | Discernment | -0.88 | 1.15 | [-3.13, 1.38] | -0.76 | 0.446 |
|  |  |  |  |  |  |  |
| Negative (Extended) | Fake | -0.55 | 0.80 | [-2.12, 1.01] | -0.69 | 0.488 |
|  | Real | 0.80 | 0.75 | [-0.67, 2.28] | 1.07 | 0.284 |
|  | Discernment | 1.36 | 1.15 | [-0.9, 3.62] | 1.18 | 0.238 |
|  |  |  |  |  |  |  |
| Arousal | Fake | 0.04 | 0.02 | [-0.01, 0.09] | 1.47 | 0.141 |
|  | Real | -0.01 | 0.02 | [-0.06, 0.03] | -0.52 | 0.604 |
|  | Discernment | -0.05 | 0.04 | [-0.12, 0.02] | -1.36 | 0.174 |

*Note.* Coefficients from one linear mixed effects model on news accuracy ratings per emotion adjective: accuracy ~ emotion adjective + news type + emotion:news type. False = beta coefficients for the correlation between increased affective state and judgment of false news; Real = beta coefficients for the correlation between increased affective state and judgment of real news; Discernment = beta coefficients for the interaction between emotion and news type (i.e., the difference between real and fake news).

**Table S5**

*Association of emotion with agreement ratings with false COVID-19 beliefs*

| **Parameter** | **Coefficient (b)** | **SE** | **95% CI** | **t** | ***p*** |
| --- | --- | --- | --- | --- | --- |
| Active | 1.99 | 0.93 | [0.15, 3.82] | 2.12 | 0.034 |
| Distressed | -1.11 | 0.94 | [-2.95, 0.73] | -1.19 | 0.235 |
| Interested | 0.63 | 0.94 | [-1.21, 2.47] | 0.67 | 0.503 |
| Excited | -0.64 | 0.94 | [-2.48, 1.2] | -0.68 | 0.494 |
| Upset | 1.23 | 0.94 | [-0.61, 3.07] | 1.31 | 0.190 |
| Strong | 1.25 | 0.94 | [-0.59, 3.09] | 1.33 | 0.183 |
| Guilty | -1.48 | 0.94 | [-3.31, 0.36] | -1.57 | 0.115 |
| Scared | 0.61 | 0.94 | [-1.24, 2.45] | 0.65 | 0.519 |
| Hostile | 0.19 | 0.94 | [-1.65, 2.03] | 0.20 | 0.840 |
| Inspired | -1.08 | 0.94 | [-2.92, 0.76] | -1.15 | 0.248 |
| Proud | 2.10 | 0.93 | [0.27, 3.93] | 2.25 | 0.024 |
| Irritable | -0.41 | 0.94 | [-2.26, 1.43] | -0.44 | 0.659 |
| Enthusiastic | 0.19 | 0.94 | [-1.65, 2.03] | 0.20 | 0.842 |
| Ashamed | -0.43 | 0.94 | [-2.27, 1.41] | -0.45 | 0.649 |
| Alert | 2.73 | 0.93 | [0.9, 4.55] | 2.93 | 0.003 |
| Nervous | -1.24 | 0.94 | [-3.08, 0.6] | -1.32 | 0.187 |
| Determined | 1.45 | 0.94 | [-0.39, 3.29] | 1.55 | 0.122 |
| Attentive | 1.01 | 0.94 | [-0.83, 2.85] | 1.07 | 0.284 |
| Jittery | -0.22 | 0.94 | [-2.06, 1.62] | -0.23 | 0.816 |
| Afraid/Anxious | -0.72 | 0.94 | [-2.56, 1.12] | -0.77 | 0.441 |
| Sad | -1.13 | 0.94 | [-2.97, 0.7] | -1.21 | 0.226 |
| Surprised | 2.15 | 0.93 | [0.32, 3.99] | 2.31 | 0.021 |
| Happy | -0.12 | 0.94 | [-1.96, 1.73] | -0.12 | 0.901 |
| Angry | 1.28 | 0.94 | [-0.56, 3.11] | 1.36 | 0.174 |
| Relaxed | 1.30 | 0.94 | [-0.53, 3.14] | 1.39 | 0.164 |
| Stressed | -2.11 | 0.93 | [-3.94, -0.28] | -2.26 | 0.024 |
| Positive | 1.32 | 0.94 | [-0.51, 3.16] | 1.41 | 0.158 |
| Negative | -0.64 | 0.94 | [-2.48, 1.2] | -0.68 | 0.495 |
| Positive (Extended) | -0.53 | 0.94 | [-2.37, 1.31] | -0.56 | 0.573 |
| Negative (Extended) | 1.27 | 0.94 | [-0.57, 3.11] | 1.35 | 0.176 |
| Arousal | 0.02 | 0.03 | [-0.04, 0.08] | 0.65 | 0.517 |

*Note.* Coefficients from linear mixed effects models on agreement ratings with COVID-19 beliefs, with one model per emotion adjective as the predictor. Emotion adjectives from the extended PANAS scale. Significance level set at α <.002 (α = .05/30) with Bonferroni correction.

## Results: Text Responses to News Items

**Figure S2***Word clouds of open text responses describing first impulses and thoughts about real (left panel, N = 2,740) and false (right panel, N = 2,873) items.*

*
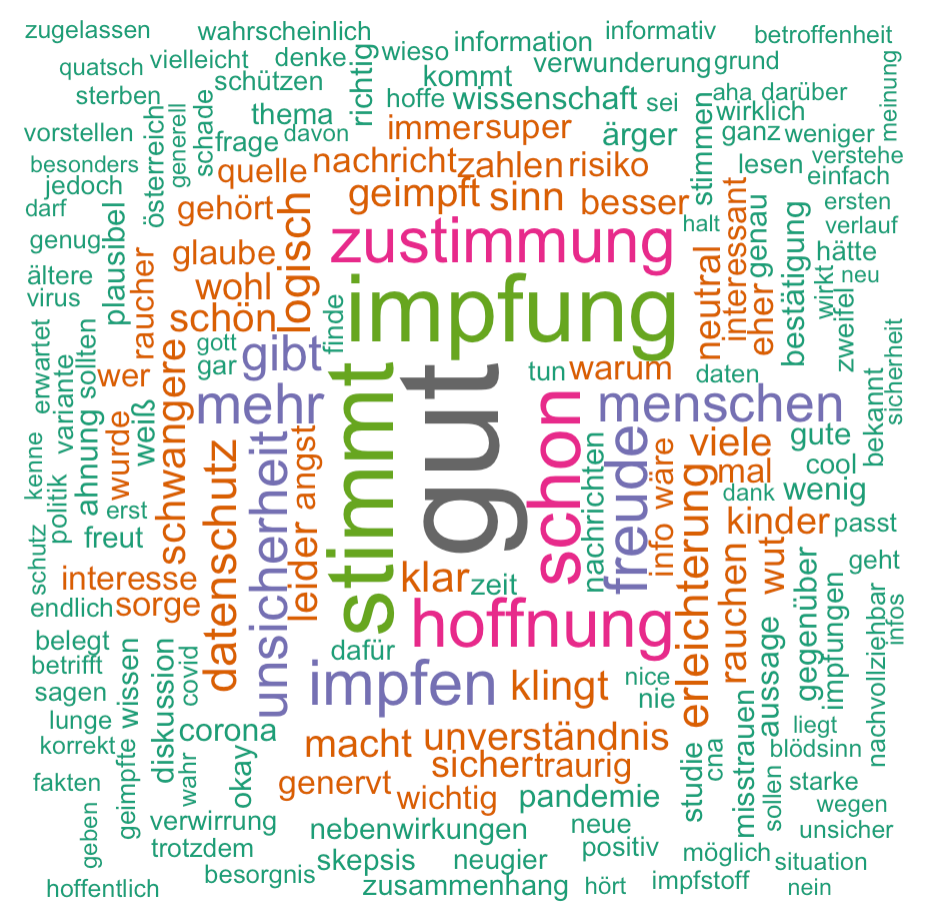
*
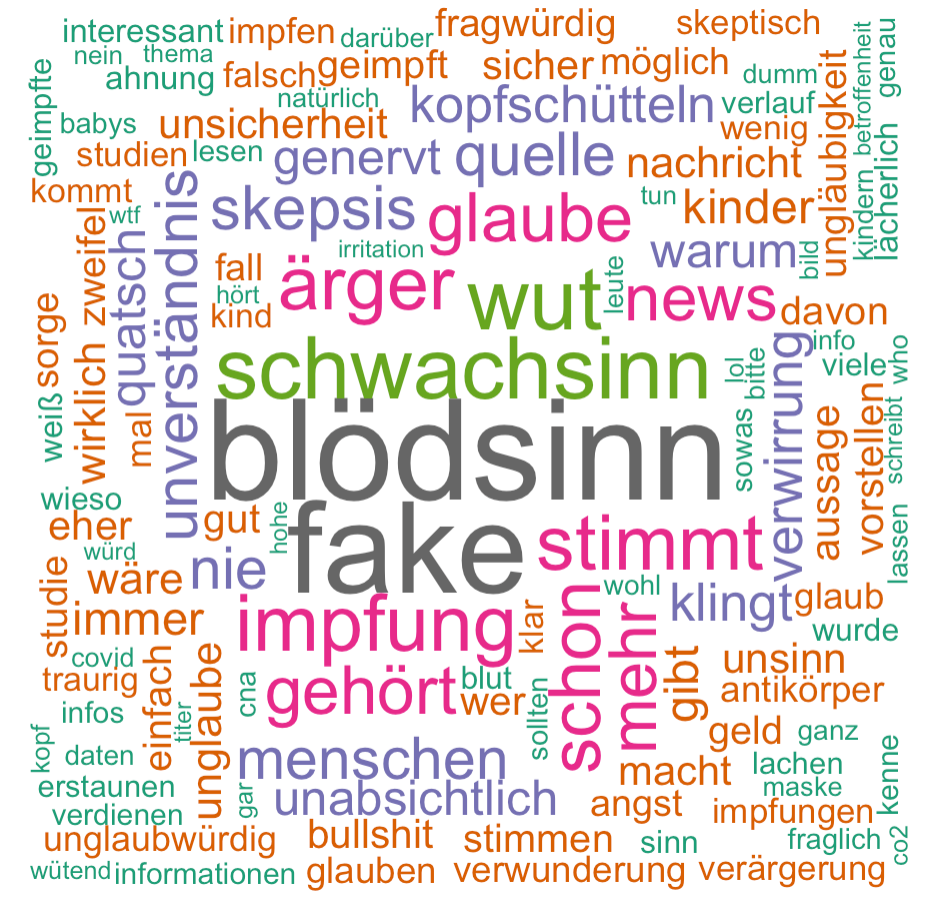


*Note.* The size of words represents the absolute frequency of words being mentioned across all participants. Angry words were among the most frequent words in text responses to false news.

**Figure S3**

*Word shift graph for open text responses describing first thoughts about false news items, comparing items that elicited angry (N = 1,500) vs. non-angry ratings (N = 1,373).*


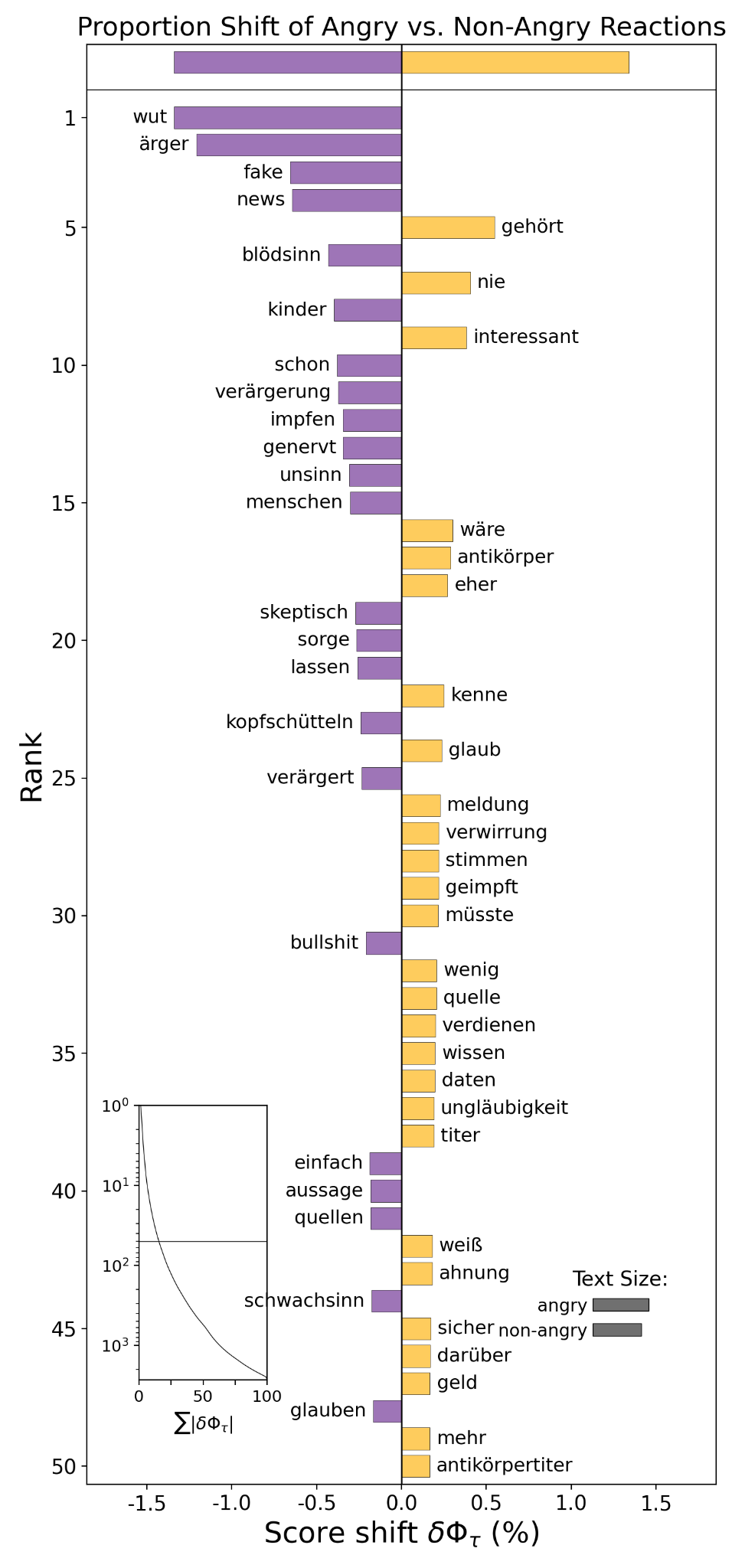


*Note*. This graph compares the proportions of words for which participants reported angry vs. non-angry emotional responses. The left side (negative score) means words were more frequent in angry reactions, whereas words on the right side (positive score) were more frequent after non-angry reactions. Words indicating “fake” (falsch) or “bullshit” (Blödsinn) were ranked highly for angry responses.

**Figure S4**

**
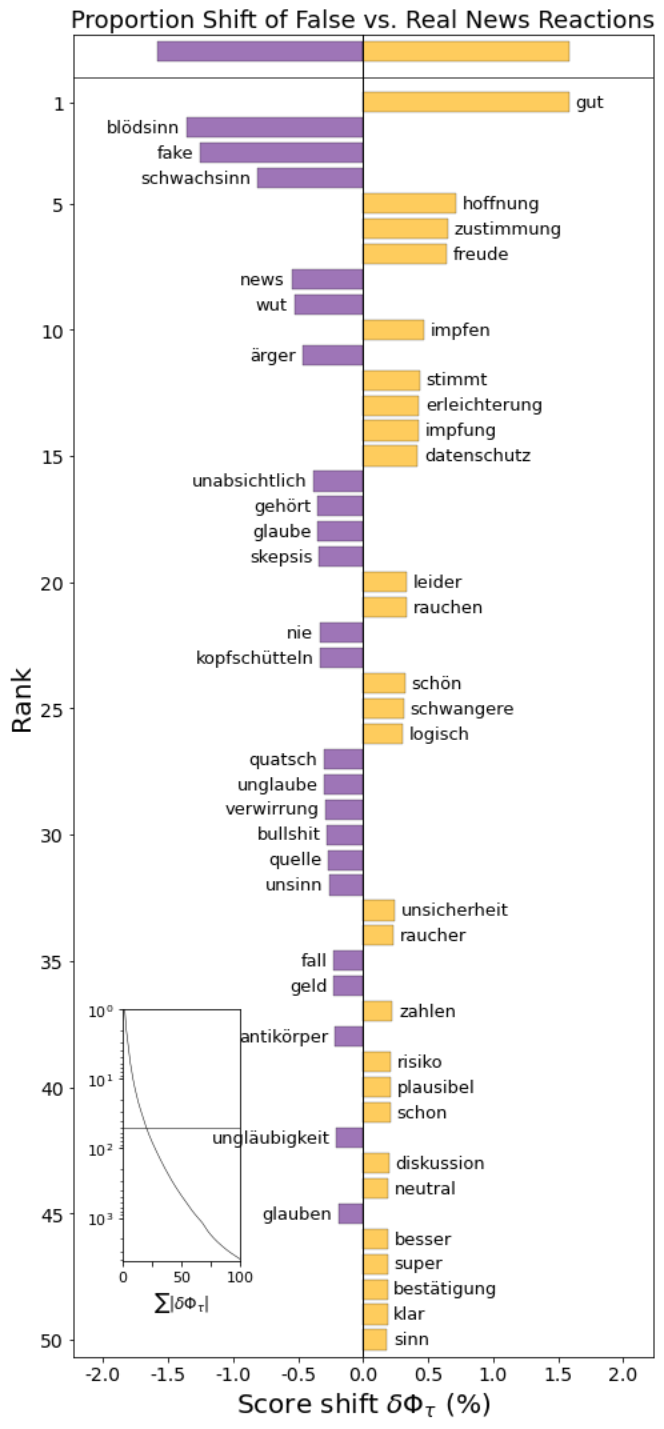
**

*Note.* Total *N* = 27,468 in words (5,613 texts). The left side (negative score) means words were more frequent after false news (*N* = 14,224), whereas words on the right side were more frequent after real news (*N* = 13,244). For English word translations, see Figure 3 in the main manuscript.

## Results: Emotional Responses and Rating Task Performance: Linear and Curvi-linear (Polynomial) Mixed Effect Models

### Anxiety

**Table S6***Linear model for anxiety, predicted by accuracy ratings for false news*

|  | **Anxiety** | | | |
| --- | --- | --- | --- | --- |
| *Predictors* | *Estimates* | *CI* | *t* | *p* |
| (Intercept) | 0.20 | 0.14 – 0.26 | 6.45 | **<0.001** |
| rating | 0.21 | 0.17 – 0.24 | 11.62 | **<0.001** |
| false | -0.20 | -0.25 – -0.16 | -8.68 | **<0.001** |
| rating * false | -0.18 | -0.23 – -0.13 | -7.21 | **<0.001** |
| **Random Effects** | | | | |
| σ^2^ | 0.76 | | | |
| τ_00_ _participant_id_ | 0.28 | | | |
| ICC | 0.27 | | | |
| N _participant_id_ | 419 | | | |
| Marginal R^2^ / Conditional R^2^ | 0.013 / 0.282 | | | |

**Table S7***Linear model for anxiety, predicted by accuracy ratings for real news*

|  | **Anxiety** | | | |
| --- | --- | --- | --- | --- |
| *Predictors* | *Estimates* | *CI* | *t* | *p* |
| (Intercept) | -0.00 | -0.06 – 0.06 | -0.06 | 0.955 |
| rating | 0.03 | -0.00 – 0.06 | 1.76 | 0.078 |
| real | 0.20 | 0.16 – 0.25 | 8.68 | **<0.001** |
| rating * real | 0.18 | 0.13 – 0.23 | 7.21 | **<0.001** |
| **Random Effects** | | | | |
| σ^2^ | 0.76 | | | |
| τ_00_ _participant_id_ | 0.28 | | | |
| ICC | 0.27 | | | |
| N _participant_id_ | 419 | | | |
| Marginal R^2^ / Conditional R^2^ | 0.013 / 0.282 | | | |

###

### Joy

**Table S8***Linear model of joy predicted by accuracy ratings for false items*

|  | **Joy** | | | |
| --- | --- | --- | --- | --- |
| *Predictors* | *Estimates* | *CI* | *t* | *p* |
| (Intercept) | -0.20 | -0.25 – -0.15 | -8.27 | **<0.001** |
| rating | 0.03 | -0.00 – 0.07 | 1.81 | 0.071 |
| false | 0.33 | 0.28 – 0.38 | 13.26 | **<0.001** |
| rating * false | 0.20 | 0.15 – 0.26 | 7.82 | **<0.001** |
| **Random Effects** | | | | |
| σ^2^ | 0.88 | | | |
| τ_00_ _participant_id_ | 0.11 | | | |
| ICC | 0.11 | | | |
| N _participant_id_ | 419 | | | |
| Marginal R^2^ / Conditional R^2^ | 0.076 / 0.177 | | | |

**Table S9**

*Linear model of joy predicted by accuracy ratings for real items*

|  | **Joy** | | | |
| --- | --- | --- | --- | --- |
| *Predictors* | *Estimates* | *CI* | *t* | *p* |
| (Intercept) | 0.13 | 0.08 – 0.18 | 5.52 | **<0.001** |
| rating | 0.24 | 0.20 – 0.27 | 13.39 | **<0.001** |
| real | -0.33 | -0.38 – -0.28 | -13.26 | **<0.001** |
| rating * real | -0.20 | -0.26 – -0.15 | -7.82 | **<0.001** |
| **Random Effects** | | | | |
| σ^2^ | 0.88 | | | |
| τ_00_ _participant_id_ | 0.11 | | | |
| ICC | 0.11 | | | |
| N _participant_id_ | 419 | | | |
| Marginal R^2^ / Conditional R^2^ | 0.076 / 0.177 | | | |

###

### Anger

**Table S10**

*Linear mixed effect model for anger predicted by accuracy ratings for false news (real is coded as baseline)*

|  | **Anger** | | | |
| --- | --- | --- | --- | --- |
| *Predictors* | *Estimates* | *CI* | *t* | *p* |
| (Intercept) | 0.18 | 0.12 – 0.23 | 5.92 | **<0.001** |
| rating | -0.18 | -0.22 – -0.15 | -10.45 | **<0.001** |
| real | -0.32 | -0.36 – -0.27 | -13.87 | **<0.001** |
| rating * false | 0.12 | 0.07 – 0.17 | 4.83 | **<0.001** |
| **Random Effects** | | | | |
| σ^2^ | 0.74 | | | |
| τ_00_ _participant_id_ | 0.25 | | | |
| ICC | 0.25 | | | |
| N _participant_id_ | 419 | | | |
| Marginal R^2^ / Conditional R^2^ | 0.064 / 0.301 | | | |

**Table S11**

*Polynomial mixed effect model for anger, predicted by accuracy ratings for false news*

|  | **Anger** | | | |
| --- | --- | --- | --- | --- |
| *Predictors* | *Estimates* | *CI* | *t* | *p* |
| (Intercept) | -0.05 | -0.11 – 0.02 | -1.36 | 0.175 |
| rating [1st degree] | -0.09 | -0.13 – -0.05 | -4.78 | **<0.001** |
| rating [2nd degree] | 0.29 | 0.25 – 0.33 | 13.12 | **<0.001** |
| false | -0.20 | -0.26 – -0.14 | -6.74 | **<0.001** |
| rating [1st degree] * false | -0.07 | -0.13 – -0.02 | -2.54 | **0.011** |
| rating [2nd degree] * false | -0.13 | -0.18 – -0.08 | -4.69 | **<0.001** |
| **Random Effects** | | | | |
| σ^2^ | 0.72 | | | |
| τ_00_ _participant_id_ | 0.25 | | | |
| ICC | 0.26 | | | |
| N _participant_id_ | 419 | | | |
| Marginal R^2^ / Conditional R^2^ | 0.085 / 0.324 | | | |

**Table S12**

*Linear model for anger, predicted by accuracy ratings for real news*

|  | **Anger** | | | |
| --- | --- | --- | --- | --- |
| *Predictors* | *Estimates* | *CI* | *t* | *p* |
| (Intercept) | -0.14 | -0.20 – -0.09 | -4.89 | **<0.001** |
| rating | -0.07 | -0.10 – -0.03 | -4.00 | **<0.001** |
| real | 0.32 | 0.27 – 0.36 | 13.87 | **<0.001** |
| rating * real | -0.12 | -0.17 – -0.07 | -4.83 | **<0.001** |
| **Random Effects** | | | | |
| σ^2^ | 0.74 | | | |
| τ_00_ _participant_id_ | 0.25 | | | |
| ICC | 0.25 | | | |
| N _participant_id_ | 419 | | | |
| Marginal R^2^ / Conditional R^2^ | 0.064 / 0.301 | | | |

**Table S13**

*Polynomial model for anger, predicted by accuracy ratings for real news*

|  | **Anger** | | | |
| --- | --- | --- | --- | --- |
| *Predictors* | *Estimates* | *CI* | *t* | *p* |
| (Intercept) | -0.25 | -0.31 – -0.18 | -7.70 | **<0.001** |
| rating [1st degree] | -0.16 | -0.20 – -0.12 | -7.88 | **<0.001** |
| rating [2nd degree] | 0.16 | 0.12 – 0.20 | 8.12 | **<0.001** |
| real | 0.20 | 0.14 – 0.26 | 6.74 | **<0.001** |
| rating [1st degree] *  real | 0.07 | 0.02 – 0.13 | 2.54 | **0.011** |
| rating [2nd degree] *  real | 0.13 | 0.08 – 0.18 | 4.69 | **<0.001** |
| **Random Effects** | | | | |
| σ^2^ | 0.72 | | | |
| τ_00_ _participant_id_ | 0.25 | | | |
| ICC | 0.26 | | | |
| N _participant_id_ | 419 | | | |
| Marginal R^2^ / Conditional R^2^ | 0.085 / 0.324 | | | |

###

### Anger with excluded outliers

We excluded three outliers that seemed to drive the curvilinear shape of the model. Model fit decreases, but remains significant when removing outliers that could explain the curvilinear tendency, χ²(2) = 96.02, *p* < .001.

**Table S14**

*Linear model for anger, predicted by accuracy ratings for false news excluding outliers*

|  | **Anger** | | | |
| --- | --- | --- | --- | --- |
| *Predictors* | *Estimates* | *CI* | *t* | *p* |
| (Intercept) | 0.15 | 0.09 – 0.21 | 5.22 | **<0.001** |
| rating | -0.20 | -0.23 – -0.16 | -11.21 | **<0.001** |
| false | -0.31 | -0.36 – -0.27 | -13.43 | **<0.001** |
| rating * false | 0.14 | 0.09 – 0.19 | 5.78 | **<0.001** |
| **Random Effects** | | | | |
| σ^2^ | 0.73 | | | |
| τ_00_ _participant_id_ | 0.22 | | | |
| ICC | 0.23 | | | |
| N _participant_id_ | 416 | | | |
| Marginal R^2^ / Conditional R^2^ | 0.067 / 0.284 | | | |

**Table S15**

*Polynomial model for anger, predicted by accuracy ratings for false news excluding outliers*

|  | **Anger** | | | |
| --- | --- | --- | --- | --- |
| *Predictors* | *Estimates* | *CI* | *t* | *p* |
| (Intercept) | -0.06 | -0.13 – 0.00 | -1.86 | 0.063 |
| rating [1st degree] | -0.10 | -0.14 – -0.07 | -5.38 | **<0.001** |
| rating [2nd degree] | 0.28 | 0.24 – 0.32 | 12.63 | **<0.001** |
| false | -0.20 | -0.26 – -0.14 | -6.69 | **<0.001** |
| rating [1st degree] *  false | -0.05 | -0.10 – 0.01 | -1.65 | 0.099 |
| rating [2nd degree] *  false | -0.13 | -0.18 – -0.07 | -4.56 | **<0.001** |
| **Random Effects** | | | | |
| σ^2^ | 0.72 | | | |
| τ_00_ _participant_id_ | 0.23 | | | |
| ICC | 0.24 | | | |
| N _participant_id_ | 416 | | | |
| Marginal R^2^ / Conditional R^2^ | 0.086 / 0.309 | | | |

###

###

### Sadness

**Table S16**

*Linear model for sadness, predicted by ratings for false news*

|  | **Sadness (false)** | | | |
| --- | --- | --- | --- | --- |
| *Predictors* | *Estimates* | *CI* | *t* | *p* |
| (Intercept) | 0.20 | 0.14 – 0.26 | 6.60 | **<0.001** |
| rating | 0.11 | 0.08 – 0.15 | 6.29 | **<0.001** |
| false | -0.28 | -0.33 – -0.24 | -12.01 | **<0.001** |
| rating * false | -0.04 | -0.09 – 0.01 | -1.52 | 0.130 |
| **Random Effects** | | | | |
| σ^2^ | 0.76 | | | |
| τ_00_ _participant_id_ | 0.28 | | | |
| ICC | 0.27 | | | |
| N _participant_id_ | 419 | | | |
| Marginal R^2^ / Conditional R^2^ | 0.012 / 0.274 | | | |

**Table S17**

*Polynomial model for sadness, predicted by ratings for false news*

|  | **Sadness (false)** | | | |
| --- | --- | --- | --- | --- |
| *Predictors* | *Estimates* | *CI* | *t* | *p* |
| (Intercept) | 0.16 | 0.09 – 0.23 | 4.63 | **<0.001** |
| rating, 2 [1st degree] | 0.13 | 0.09 – 0.16 | 6.38 | **<0.001** |
| rating, 2 [2nd degree] | 0.05 | 0.00 – 0.09 | 2.17 | **0.030** |
| false | -0.30 | -0.36 – -0.24 | -9.88 | **<0.001** |
| rating, 2 [1st degree] *  false | -0.11 | -0.17 – -0.05 | -3.79 | **<0.001** |
| rating, 2 [2nd degree] *  false | 0.04 | -0.01 – 0.10 | 1.57 | 0.116 |
| **Random Effects** | | | | |
| σ^2^ | 0.76 | | | |
| τ_00_ _participant_id_ | 0.28 | | | |
| ICC | 0.27 | | | |
| N _participant_id_ | 419 | | | |
| Marginal R^2^ / Conditional R^2^ | 0.014 / 0.279 | | | |

**Table S18**

*Linear model for sadness, predicted by ratings for real news*

|  | **Sadness (real)** | | | |
| --- | --- | --- | --- | --- |
| *Predictors* | *Estimates* | *CI* | *t* | *p* |
| (Intercept) | -0.08 | -0.14 – -0.02 | -2.54 | **0.011** |
| rating | 0.08 | 0.04 – 0.11 | 4.46 | **<0.001** |
| real | 0.28 | 0.24 – 0.33 | 12.01 | **<0.001** |
| rating * real | 0.04 | -0.01 – 0.09 | 1.52 | 0.130 |
| **Random Effects** | | | | |
| σ^2^ | 0.76 | | | |
| τ_00_ _participant_id_ | 0.28 | | | |
| ICC | 0.27 | | | |
| N _participant_id_ | 419 | | | |
| Marginal R^2^ / Conditional R^2^ | 0.012 / 0.274 | | | |

**Table S19**

*Polynomial model for sadness, predicted by ratings for real news*

|  | **Sadness (real)** | | | |
| --- | --- | --- | --- | --- |
| *Predictors* | *Estimates* | *CI* | *t* | *p* |
| (Intercept) | -0.14 | -0.20 – -0.07 | -4.13 | **<0.001** |
| rating, 2 [1st degree] | 0.02 | -0.03 – 0.06 | 0.74 | 0.459 |
| rating, 2 [2nd degree] | 0.09 | 0.05 – 0.13 | 4.69 | **<0.001** |
| real | 0.30 | 0.24 – 0.36 | 9.88 | **<0.001** |
| rating, 2 [1st degree] *  real | 0.11 | 0.05 – 0.17 | 3.79 | **<0.001** |
| rating, 2 [2nd degree] *  real | -0.04 | -0.10 – 0.01 | -1.57 | 0.116 |
| **Random Effects** | | | | |
| σ^2^ | 0.76 | | | |
| τ_00_ _participant_id_ | 0.28 | | | |
| ICC | 0.27 | | | |
| N _participant_id_ | 419 | | | |
| Marginal R^2^ / Conditional R^2^ | 0.014 / 0.279 | | | |

**Table S20**

*Linear model for sadness, predicted by ratings for false news excluding an outlier*

|  | **Sadness (false)** | | | |
| --- | --- | --- | --- | --- |
| *Predictors* | *Estimates* | *CI* | *t* | *p* |
| (Intercept) | 0.19 | 0.13 – 0.25 | 6.31 | **<0.001** |
| rating | 0.10 | 0.07 – 0.14 | 5.83 | **<0.001** |
| false | -0.28 | -0.32 – -0.23 | -11.82 | **<0.001** |
| rating * false | -0.03 | -0.07 – 0.02 | -1.01 | 0.312 |
| **Random Effects** | | | | |
| σ^2^ | 0.76 | | | |
| τ_00_ _participant_id_ | 0.26 | | | |
| ICC | 0.26 | | | |
| N _participant_id_ | 418 | | | |
| Marginal R^2^ / Conditional R^2^ | 0.012 / 0.265 | | | |

**Table S21**

*Polynomial model for sadness, predicted by ratings for false news excluding an outlier*

|  | **Sadness (false)** | | | |
| --- | --- | --- | --- | --- |
| *Predictors* | *Estimates* | *CI* | *t* | *p* |
| (Intercept) | 0.16 | 0.09 – 0.23 | 4.61 | **<0.001** |
| rating [1st degree] | 0.11 | 0.07 – 0.15 | 5.74 | **<0.001** |
| rating [2nd degree] | 0.04 | -0.01 – 0.08 | 1.66 | 0.097 |
| false | -0.30 | -0.36 – -0.24 | -9.98 | **<0.001** |
| rating [1st degree] *  false | -0.09 | -0.15 – -0.03 | -3.17 | **0.002** |
| rating [2nd degree] *  false | 0.05 | -0.00 – 0.11 | 1.84 | 0.066 |
| **Random Effects** | | | | |
| σ^2^ | 0.76 | | | |
| τ_00_ _participant_id_ | 0.27 | | | |
| ICC | 0.26 | | | |
| N _participant_id_ | 418 | | | |
| Marginal R^2^ / Conditional R^2^ | 0.013 / 0.270 | | | |

## Emotional Responses and Agreement with False Beliefs about COVID-19

### Statistical Analysis

We ran a linear mixed-effect model predicting each of the four emotional responses from false beliefs, news type, and their interaction (e.g. anger ~ false beliefs * news type + 1|participants), including participants as a random intercept. We included no random intercept for false belief items because the models did not converge.

**Table S22**

*Regressing false COVID-19 beliefs (for each news type), news type and their interaction on each emotional response*

| Predictor | *Anger* | *Anxiety* | *Joy* | *Sadness* |
| --- | --- | --- | --- | --- |
| False beliefs (for false news) | -0.09* | 0.12*** | 0.03 | 0.12** |
| False beliefs (for real news) | 0.12*** | 0.04 | -0.08*** | 0.05 |
| News type | 0.68*** | 0.06** | -0.42*** | 0.19*** |
| Interaction of false beliefs*news type | -0.21*** | 0.08*** | 0.11*** | 0.07** |

*Note.* **p* < .05; ***p* < .01; and ****p* < .001. We ran a separate linear mixed effects model on each emotional response (emotional response ~ false beliefs + news type + false beliefs x news type + (1/participant)). Beta-coefficients for the predictor false beliefs are reported once for each news type defined as the baseline level.

## Questionnaires, Tasks, and Text of Items

Several of the questionnaires in the list below were not analyzed for the current study, but only in projects of the students who collected the data. All items of relevance to the current manuscript are marked with an asterisk, and their text is reported below. All other items can be found on https://osf.io/tgzxr/?view_only=b4db898e32044c8ea4fbc18c4cfcc1ab (except for published questionnaires).

### Order of questionnaires and tasks

1. Socio-demographics: gender, age, minority affiliation*, political orientation (left-right)*, highest education*, monthly household income, occupation
2. Personal attitudes about the COVID-19 situation and counter-measures*
3. Need for Cognition (NfC; Beißert et al., 2015)
4. 7-items Cognitive Reflection Test (CRT; translated by Weiss et al., 2021)
5. 4-items Need for Uniqueness (NFU; Lynn & Harris, 1997, translated by Ingendahl et al., 2021)
6. Authoritarianism Short Scale (KSA-3; Nießen et al., 2019)
7. Tolerance for Ambiguity (AM; Herman et al., 2010, translated by authors)
8. Prior affective state: 20-item Positive and Negative Affect Schedule scale (PANAS; Breyer & Bluemke, 2016) and 6 additional items: sad, surprised, happy, angry, relaxed and stressed*
9. Agreement with 9 COVID-19 beliefs (rating of 8 false and 1 real statement)*
10. COVID-19 news rating task and emotional response for 24 items (12 false)*
11. Vaccination Status (yes/no)*
12. Social Media Use: Social media platforms used to get the latest news and perceived credibility of these media
13. Twitter Use (yes/no) & Handle: Request to share handle for research purposes + data use consent statement

### Socio-demographics

1. Minority affiliation: Wenn Sie Teil einer ethnischen, religiösen, sexuellen oder anderen Minderheit sind. Welcher Minderheit gehören Sie an? (If you are part of an ethnic, religious, sexual or other minority, which minority do you belong to?)
   1. Ethnische oder Nationale Minderheit (Ethnic or national minority)
   2. Sprachliche Minderheit (Linguistic minority)
   3. Religiöse Minderheit (Religious minority)
   4. Minderheit sexueller Orientierung (Sexual minority)
   5. Soziale Minderheit (z.B. aufgrund finanzieller Benachteiligung) (Social minority; E.g. due to financial disadvantage)
   6. andere Minderheit (Other minority)
2. Education: Bitte geben Sie Ihre höchste abgeschlossene Ausbildung an (Please indicate your highest completed education)
   1. Pflichtschule (Compulsory schooling, 9 years in Austria)
   2. Lehre (Apprenticeship)
   3. Matura (High school diploma)
   4. Hochschulabschluss (University degree)
3. Political Orientation: Wie würden Sie Ihre politische Orientierung beschreiben? (How would you describe your political orientation?):
   1. Links (Left)
   2. Mitte-links (Center-left)
   3. Mitte (Center)
   4. Mitte-richts (Center-right)
   5. Rechts (Right)

###

### Agreement with false COVID-19 Beliefs

1. Kennen Sie diese Aussage? (Do you know this statement?)
   - Ja (yes)
   - Ich glaube, ich habe so etwas schon einmal gehört bzw. gelesen. (I think I have heard or read something like this before.)
   - Nein (no)
2. Agreement with 9 statements: Auf einer Skala von 0% bis 100%: Wie sehr stimmen Sie zu, dass diese Aussage wahr ist? (On a scale from 0-100%: How much do you agree that this statement is true?)

| **Statements about vaccines (5), masks (1), PCR-tests (1), and natural immune response (2)** | **Type** |
| --- | --- |
| 1. Gegen Covid-19 geimpfte Personen sind genauso ansteckend wie ungeimpfte Personen. (Persons vaccinated against Covid-19 are as contagious as unvaccinated persons.) | false |
| 1. Corona Impfung bringt mehr Todesfälle als andere Impfungen.  (Corona vaccination causes more deaths than other vaccinations) | false |
| 1. Basische Lebensmittel helfen gegen Corona.  (Alkaline foods help against Corona.) | false |
| 1. Das Langzeitrisiko von Impfungen ist nicht ausreichend erforscht. (The long-term risk of vaccinations has not been adequately researched.) | false |
| 1. Masken gefährden die Gesundheit von Kindern. (Masks put children's health at risk.) | false |
| 1. Ungeimpfte haben ein höheres Risiko für einen schweren Verlauf. (Unvaccinated have a higher risk of a severe course of COVID-19.) | real |
| 1. Ein gesundes Immunsystem schützt vor einer Corona Erkrankung. (A healthy immune system protects against Corona disease.) | false |
| 1. Die Corona Impfung macht Frauen unfruchtbar. (Corona vaccination makes women infertile.) | false |
| 1. In Gurgeltests befinden sich kleine Metalle, die die Schleimhäute anritzen. (There are small metals in gargle tests that score the mucous membranes.) | false |

###

### COVID-19 news rating task and emotional response

For each news item, participants answered the following items:

1. Open text field: Welchen Impuls oder Gedanken löst diese Nachricht bei Ihnen aus?
   (Which impulse or thought does this news trigger in you?)
2. Familiarity rating: Haben Sie den Inhalt dieser Nachricht schon einmal gelesen oder gehört? (Have you ever read or heard the contents of this message?)
   1. Ja (yes)
   2. Ich glaube, ich habe so etwas schon einmal gehört bzw. gelesen. (I think I have heard or read something like this before.)
   3. Nein (no)
3. News accuracy rating, visual analogue scale 0-100%: Nach Ihrem besten Wissen und Gewissen, wie zutreffend ist diese Nachricht? (To the best of your knowledge, how accurate is this news item?)
4. Confidence, visual analogue scale 0-100%: Wie sicher sind Sie sich, dass ihre Einschätzung stimmt? (How confident are you that their assessment is correct?)
5. Emotional response (6-Point Likert scale):
   Gar nicht, schwach, eher schwach, mittelmäßig, eher stark, stark
   (Not at all, weak, rather weak, moderate, rather strong, strong)
   1. Overall: Löst diese Nachricht ein Gefühl aus bei Ihnen? Ja/Nein (Does this news trigger any emotion in you? Yes/No)
   2. Four basic emotions: Wenn ich diese Nachricht lese, empfinde ich: (When I read this news, I feel:)
      - Verärgert, wütend (annoyed, angry)
      - Traurig, betroffen (sad, concerned)
      - Freudig, aufgeregt (joyful, excited)
      - Verängstigt, unsicher (fearful, uncertain)
      - Anderes Gefühl (another feeling)

### Vaccination status

Haben Sie sich gegen das Coronavirus impfen lassen? (Have you been vaccinated against the coronavirus?)

- Ja (Yes)
- Nein (No)

## References

Beißert, H., Köhler, M., Rempel, M., & Beierlein, C. (2015). Deutschsprachige Kurzskala zur Messung des Konstrukts Need for Cognition NFC-K. *Zusammenstellung sozialwissenschaftlicher Items und Skalen (ZIS)*. https://doi.org/10.6102/ZIS230

Breyer, B., & Bluemke, M. (2016). Deutsche Version der Positive and Negative Affect Schedule PANAS (GESIS Panel). *Zusammenstellung sozialwissenschaftlicher Items und Skalen (ZIS)*. https://doi.org/10.6102/ZIS242

Herman, J. L., Stevens, M. J., Bird, A., Mendenhall, M., & Oddou, G. (2010). The Tolerance for Ambiguity Scale: Towards a more refined measure for international management research. *International Journal of Intercultural Relations*, *34*(1), 58–65. https://doi.org/10.1016/j.ijintrel.2009.09.004

Ingendahl, M., Hummel, D., Maedche, A., & Vogel, T. (2021). Who can be nudged? Examining nudging effectiveness in the context of need for cognition and need for uniqueness. *Journal of Consumer Behaviour*, *20*(2), 324–336. https://doi.org/10.1002/cb.1861

Lynn, M., & Harris, J. (1997). Individual Differences in the Pursuit of Self-Uniqueness Through Consumption. *Journal of Applied Social Psychology*, *27*(21), 1861–1883. https://doi.org/10.1111/j.1559-1816.1997.tb01629.x

Nießen, D., Schmidt, I., Beierlein, C., & Lechner, C. M. (2019). Authoritarianism Short Scale (KSA-3). *ZIS - The Collection of Items and Scales for the Social Sciences*. https://doi.org/10.6102/ZIS272

Weiss, A., Dorrough, A. R., & Schmitz, L. (2021). Analytic atheism in a low-religiosity culture: Examining the relationship between analytic thinking and religious belief in Germany. *Personality and Individual Differences*, *178*, 110854. https://doi.org/10.1016/j.paid.2021.110854
